# Coupling coordination between agricultural carbon emission efficiency and food security in China: The spatial-temporal evolution and prediction

**DOI:** 10.1371/journal.pone.0325026

**Published:** 2025-07-02

**Authors:** Xixian Zheng, Wenmei Liao

**Affiliations:** 1 School of Economics and Management, Jiangxi Agricultural University, Nanchang, China; 2 Research Center for the Three Rural Issues, Jiangxi Agricultural University, Nanchang, China; 3 Institute of Rural Economy, Jiangxi Agricultural University, Nanchang, China; University of Jaen: Universidad de Jaen, SPAIN

## Abstract

Addressing climate change and food insecurity is crucial for achieving the Sustainable Development Goals (SDGs), with the enhancement of Agricultural Carbon Emission Efficiency (ACEE) and the assurance of Food Security (FS) playing pivotal roles. This study conducts a comprehensive evaluation of the coordination between ACEE and FS across 31 Chinese provinces from 2007 to 2021. The methodology employs the Super Slack-Based Model (SBM) and the Entropy- Weighted Technique for Order Preference by Similarity to Ideal Solution (EW-TOPSIS) for ACEE and FS assessments, respectively, while utilizing the Coupling Coordination Degree Model (CCDM) to gauge their coordination. This research applies Global spatial autocorrelation (GA), Local Indicators of Spatial Association (LISA) clustering, and Markov chain analysis to investigate the spatial correlation characteristics and dynamic evolutionary probability of Coupling Coordination Degree (CCD). Additionally, a Combination Forecasting Model predicts CCD trends through 2030. The findings indicate positive trends in both ACEE and FS, albeit with significant regional disparities and a notable lag of FS behind ACEE improvement. The evolution of China’s CCD from “barely coordinated” towards “primary coordination” is evident, with the northeastern and central regions outperforming their western and eastern counterparts. Spatial analysis reveals pronounced clustering of CCD, indicating the impact of spatial spillover effects. Markov chain analysis shows that provinces have at least a 71.4% probability of maintaining their current CCD status, revealing substantial system inertia. Projections point to increasing CCD levels across various regions, with the northeastern region expected to reach 0.827 by 2030 (a 5.72% increase from 2021), followed by the central (0.742), western (0.659), and eastern regions (0.638); however, regional imbalances persist, highlighting the need for concentrated efforts in areas experiencing CCD stagnation or decline. The study concludes with a call for region-specific development strategies, enhanced resource allocation and technological support, and a comprehensive policy framework that accounts for the long-term and spatial dimensions, aiming to facilitate equitable advancements in reducing agricultural carbon emissions and enhancing food security.

## 1. Introduction

Achieving zero hunger and addressing climate change constitute fundamental objectives within the United Nations Sustainable Development Goals (SDGs) framework [1]. The persistent progression of climate change presents a substantial threat to terrestrial ecosystems, precipitating biodiversity loss, soil degradation, and water pollution [2]. Consequently, reducing carbon emissions and enhancing carbon emission efficiency have become imperative for climate change mitigation strategies.

The challenges of climate change mitigation and food security are further compounded by global population growth and accelerating urbanization. The world population approximated 8.2 billion by mid-2024 and is projected to increase to approximately 10.3 billion by the mid-2080s, with the majority of this growth concentrated in developing regions. Concurrently, global urbanization has intensified, with urban populations expanding from 751 million in 1950 to 4.4 billion in 2020, constituting 56.2% of the global population. This proportion is anticipated to reach 68% by 2050. This rapid urbanization has led to significant land-use changes worldwide. A recent study quantifying urban expansion from 1985 to 2018 in major global cities revealed that urban areas have expanded by 80% during this period, primarily encroaching upon agricultural lands [3]. This expansion not only reduces arable land availability but also increases the distance between food production and consumption centers, leading to higher carbon emissions from agricultural transportation and more complex food supply chains.

Agriculture functions as both a carbon source and sink, thereby playing a crucial role in carbon emission mitigation strategies. Currently, global agricultural carbon emissions constitute approximately 11% of total emissions, with developing nations contributing disproportionately to this figure [4]. The Chinese government’s established targets for achieving carbon peak by 2030 and carbon neutrality by 2060 underscore the imperative of implementing low-carbon agricultural practices. Furthermore, the COVID-19 pandemic has exposed vulnerabilities within agricultural food systems, demonstrating their limited resilience to external shocks and highlighting critical weaknesses in food supply chains. As the world’s most populous developing nation, China utilizes merely 9% of global arable land to generate 25% of the world’s food supply, thereby sustaining approximately one-fifth of the global population [5]. Given the foundational importance of food security to societal development, the Chinese government accords high priority to Food Security (FS) within its policy framework. In comparison with developed nations, China faces more significant challenges in carbon emission reduction while simultaneously experiencing heightened vulnerability to food shortage risks. Consequently, Emission Efficiency (ACEE) and FS has become imperative for sustainable development. This study investigates the coordinated relationship between ACEE and FS, analyzing their spatial-temporal evolution patterns and projecting future trajectories of their relationship.

This study makes several substantive theoretical and methodological contributions beyond merely applying existing frameworks to new contexts. First, while prior research on food security has predominantly concentrated on the supply dimension, this study advances theory by proposing an integrated conceptual framework that systematically connects four critical dimensions of food security (supply security, access security, production stability, and production sustainability). This multi-dimensional approach transcends the traditional water-energy-food nexus studies by explicitly incorporating sustainability and stability dimensions that capture both short-term resilience and long-term ecological viability. Second, this research develops a novel theoretical perspective by conceptualizing the ACEE-FS relationship not as a unidirectional or independent system, but as dynamically coupled subsystems with complex feedback mechanisms. While coupling coordination models have been applied in various contexts, their application to the ACEE-FS relationship represents an important theoretical extension that illuminates how these seemingly competing objectives can be understood as synergistic elements of a unified sustainable development framework. Third, our methodological innovation lies in the development of an integrated spatial-temporal analytical framework that combines multiple complementary approaches (Super-SBM, EW-TOPSIS, CCDM, spatial analysis, and combination forecasting) to capture not only the current state but also the evolutionary trajectories and future trends of this complex coupled system.

## 2. Literature review

### 2.1. Agricultural carbon emission efficiency (ACEE) and its measurement

Agricultural carbon reduction constitutes a prominent topic in contemporary academic discourse. The concept of ACEE has been developed as a pragmatic analytical framework for evaluating carbon emission reduction potential and assessing the efficacy of green agricultural development initiatives [6]. Existing literature has concentrated on measuring ACEE [7], investigating regional disparities [8], identifying influencing factors [9], and implementing analyses across diverse geographical contexts, including China at the national scale [10], the Yellow River Basin [11] and the Changjiang Economic Corridor [12].

The precise quantification of ACEE is critically important. Historically, researchers have employed “single factor” methodologies to estimate ACEE, such as carbon productivity [13], and carbon indices [14]. While these single factor indices offer computational simplicity, they exhibit significant limitations in capturing the multidimensional characteristics of carbon emission efficiency and its complex interactions with environmental and social variables. Consequently, researchers have advanced evaluation methodologies by incorporating the Entropy Method [15], Analytic Hierarchy Process (AHP) [16], and Data Envelopment Analysis (DEA) [17] for ACEE assessment. Among these analytical approaches, the DEA model has emerged as the most extensively utilized framework for evaluating carbon emission efficiency. Nevertheless, the conventional DEA model demonstrates inherent limitations in accommodating undesirable outputs. To address the influence of external environmental factors, numerous scholars have adopted the Data Envelopment Analysis Slack-Based Measure (DEA-SBM) model [18] and the Super-SBM model [19], both of which are widely implemented in the quantification of carbon emission efficiency. Given the critical importance of incorporating undesirable output, specifically carbon emissions generated from agricultural activities, this investigation employs the Super-SBM model for comprehensive ACEE assessment.

### 2.2. Food security (FS) and its assessment

The conceptualization of food security has undergone substantial evolution since its initial introduction into policy discourse in the early 1970s. Subsequent decades have witnessed the expansion of the food security concept to encompass four foundational pillars: availability, access, utilization, and stability [20]. Scholarly investigations of food security have addressed various dimensions, including conceptual definition [21], methodological assessment [22], and the examination of determinants [23].

The development of a comprehensive food security evaluation framework, integrating indicators across multiple perspectives, is essential for accurately assessing food security status and providing empirical support for policy formulation. While some researchers have employed singular metrics for food security assessment, such as the prevalence of malnutrition [24], and food self-sufficiency rates [25], such measures have inadequately captured the multidimensional nature of food security within the context of sustainable agricultural development. The evaluation of food security transcends the mere availability of sufficient food supply; it necessarily encompasses factors such as transport accessibility, food production stability, and food production sustainability. This research synthesizes the Food and Agriculture Organization of the United Nations (FAO) definition of food security, proposing an enhanced evaluation system that incorporates food supply security, food access security, food production stability, and food production sustainability. Within this framework, food supply security primarily addresses the adequacy of food availability to satisfy basic consumption requirements, quantified through metrics such as “grain sowing area” and “per capita grain ownership” [20]. Furthermore, food access security is evaluated through indicators such as “road network density”, “rural per capita disposable income” and the “rural Engel’s coefficient” [26]. The study also examines food production stability, which reflects the capacity to withstand natural and market-induced fluctuations, measured through metrics such as “grain yield volatility”, “grain price volatility”, and “crop disaster ratio” [27]. Lastly, food production sustainability, a crucial determinant of long-term developmental viability at the provincial level, is assessed using the “fertilizer application quantity” and “quantity of pesticide usage” [28].

### 2.3. The relationship between ACEE and FS

The relationship between agricultural carbon reduction and food security exhibits multidimensional complexity. Certain studies have identified synergistic potential between these objectives, demonstrating that the concurrent enhancement of agricultural yields and reduction of carbon emissions is achievable through technological innovations [29]. Conversely, conventional yield-augmentation practices, such as arable land expansion and intensified fertilizer application, may exacerbate carbon emissions, while specific emission reduction strategies, including the diminution of agricultural inputs, potentially compromise food productivity [30]. The Chinese government has vigorously promoted sustainable development strategies, targeting carbon peak by 2030 and carbon neutrality by 2060, while simultaneously preserving food security. A comprehensive examination of the coupling and coordination relationship between ACEE and FS will contribute significantly to the theoretical framework for integrating food security with low-carbon development, thereby facilitating the realization of coordinated sustainable development. However, extant research has predominantly focused on the interactions among water, energy, food [31], or investigated unidirectional or isolated aspects of the ACEE and FS relationship [32], thereby neglecting the comprehensive interactions inherent within these systems. Considering the protracted interplay between the food and carbon systems, investigating the coupling and coordination dynamics between ACEE and FS, and forecasting their future trajectories, becomes imperative for informed policy formulation.

In recent years, scholars across various disciplines have employed various methodologies and models to assess the coupling relationship between systems. These include Grey Relational Analysis Model [33], Fuzzy Comprehensive Evaluation Method [34], and Support Vector Machine Method [35]. Among these analytical frameworks, the Coupling Coordination Degree model (CCDM) distinguishes itself through its emphasis on the interactions between different systems or indicators, effectively capturing the extent of coordination within these interactions. This model efficaciously reveals the level of coordinated development among subsystems, and has been extensively applied across diverse fields including physics [36], ecology [37] and economics [38]. Considering the evident coupling relationship between ACEE and FS, this study utilizes the CCDM to explore the intricate ACEE-FS relationship.

In forecasting CCD, predominant research methodologies encompass the Autoregressive Integrated Moving Average (ARIMA) [39], the Grey Model (GM(1,1)) [40], and the Long Short-Term Memory (LSTM) [41]. Notably, the GM(1,1) model is characterized by its minimal data requirements and superior fitting accuracy. The ARIMA model, conversely, is distinguished by its adaptability to diverse data characteristics, rendering it particularly suitable for non-stationary time series analysis. The LSTM model, equipped with self-learning and adaptation capabilities, excels in addressing complex nonlinear challenges, offering precise and scientifically robust predictions. It has been extensively implemented in environmental modeling [42] and energy forecasting applications [43]. While the majority of investigations have employed single forecasting model, a combination forecasting approach leverages the comprehensive insights derived from individual models to enhance predictive accuracy. Consequently, this study employs a combination forecasting model to predict the CCD trajectories.

This research investigates the synergistic relationship between agricultural low-carbon development and food security across 31 provinces in China spanning the period from 2007 to 2021. Initially, it employs the Super-SBM model and the EW-TOPSIS method to evaluate the ACEE and FS, respectively. Subsequently, the CCDM is applied to assess their coordinated relationship. Furthermore, this study incorporates GA, LISA clustering, and the Markov chain analysis to explore the spatial correlation attributes and the dynamic evolutionary probabilities of CCD. Finally, the combination forecasting model is implemented to predict future CCD trends. The findings aim to provide empirical evidence supporting the synchronization of ACEE and FS in China and other developing nations, thereby facilitating the formulation of policies concerning sustainable development, carbon emission reduction, and food security enhancement.

## 3. Materials and methods

### 3.1. Study area

This investigation encompasses 31 mainland Chinese provinces over the period from 2007 to 2021 (due to data availability constraints, Hong Kong, Taiwan, and Macau are excluded from the analysis). China exhibits considerable regional disparities in terms of economic development and policy implementation. These disparities significantly influence agricultural carbon emission efficiency, food security, and their coupling coordination dynamics. To comprehensively examine the synergistic development patterns across heterogeneous regions, this study categorizes China’s economic landscape into four distinct geographical regions: eastern, western, central, and northeastern (see Table 1).

**Table 1:** Division of the four major regions in China.

Regional classification	Provinces
Eastern region	Beijing, Tianjin, Hebei, Shandong, Shanghai, Jiangsu, Zhejiang, Fujian, Guangdong, Hainan
Central region	Shanxi, Henan, Anhui, Hubei, Hunan, Jiangxi
Western region	Inner Mongolia, Shaanxi, Ningxia, Gansu, Qinghai, Xinjiang, Tibet, Sichuan, Chongqing, Guizhou, Yunnan, Guangxi
Northeastern Region	Heilongjiang, Jilin, Liaoning

### 3.2. Data sources

All empirical data for this research were acquired from official Chinese statistical repositories, including the China Statistical Yearbook (National Bureau of Statistics, 2008–2022), China Rural Statistical Yearbook (National Bureau of Statistics, 2008–2022), China Energy Statistical Yearbook (National Bureau of Statistics, 2008–2022), and the respective provincial statistical yearbooks (2008–2022). These publications provided comprehensive and authoritative data for the 31 mainland Chinese provinces throughout the study period (2007–2021).

Specifically, the China Statistical Yearbook furnished data pertaining to cultivated land resources (sown area of crops), labor inputs (population employed in primary industry), agricultural mechanization (total power of agricultural machinery), grain production parameters (grain sowing area, per capita grain ownership), and rural socioeconomic indicators (rural per capita disposable income, rural Engel’s coefficient). The China Rural Statistical Yearbook provided agricultural input data, including fertilizer application, pesticide usage, agricultural film utilization, irrigation infrastructure (effective irrigation area), agricultural vulnerability indicators (crop disaster ratio), and quantitative measures of agricultural chemical inputs (fertilizer/pesticide application quantities). Energy consumption metrics, particularly diesel fuel utilization for agricultural operations, were extracted from the China Energy Statistical Yearbook. Provincial-level data not comprehensively documented in national statistical compilations were supplemented with corresponding information from the respective provincial statistical yearbooks.

### 3.3. Methodology

**Fig 1:**
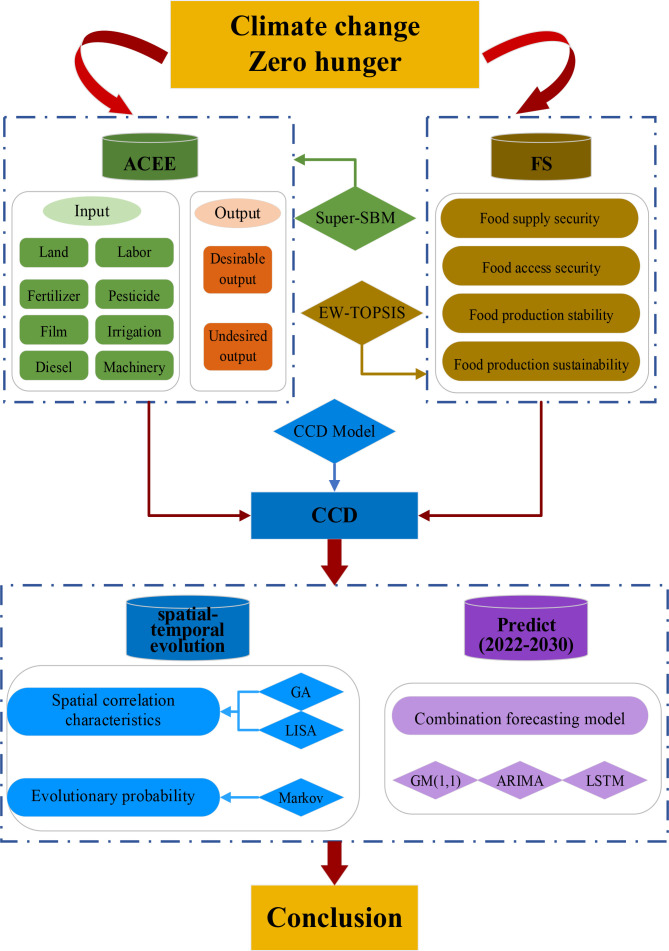
The framework of research method.

This research incorporates five primary analytical components to facilitate a comprehensive examination: (1) Evaluation of ACEE for each province utilizing the Super-SBM model; (2) Assessment of FS levels across provinces employing the EW-TOPSIS method; (3) Analysis of the coupling coordination relationship through the CCD model; (4) Investigation of spatial-temporal distribution characteristics and evolutionary probability of CCD using GA, LISA clustering, and the Markov chain methodologies; (5) Projection of CCD developmental trajectories utilizing a combination forecasting model. Fig 1 illustrates the comprehensive research framework.

#### 3.3.1. Agricultural carbon emission efficiency measurement.

Agricultural practices generate essential outputs, predominantly food products, while simultaneously presenting environmental challenges, including the emission of greenhouse gases from various agricultural activities [16]. To quantify the efficiency of carbon emissions in agriculture systems across China’s 31 provincial administrative regions, this study implements the Super-SBM model, which has the distinctive feature of incorporating undesirable outputs in its analytical framework. The mathematical formulation of the model is as follows:


$$P=\frac{\frac{1}{m}\sum_{i=1}^{m}\;\overset{―}{x_{i}}/x_{io}}{\frac{1}{s1+s_{2}}\left(\sum_{r1}^{S1}\;\overset{―}{y_{r}^{g}}\right)/y_{m}^{g}+\sum_{a=1}^{S2}\;\overset{―}{y_{a}^{b}}/y_{ao}^{g}}$$ (1)

$$\left\{ \begin{array}{c} \bar{x}\geq \sum_{j=1,j\neq 0}^{n}\;\lambda_{j}x_{j},{\bar{y}}_{r}^{g}\leq \sum_{j=1,j\neq 0}^{n}\;\lambda_{jj}^{g},{\bar{y}}_{a}^{b}\leq \sum_{j=1,j\neq 0}^{n}\;\lambda_{j}y_{aj}^{b}\\ \;\;\;\;\;\;\;\;\;\;\;\;\;\;\;\;\;\;\;\;\;\;\;\;\;\;\;\;\;\;\;\;\;\;\;\;\;\;\;\;\;\;\;\;\;\;\;\;\;\;\;\;\;\;\;\;\;\;\;\;\;\;\;\;\;\;\;\;\;\;\;\;\;\;\;\bar{x}\geq x_{io},{\bar{y}}_{r}^{g}\leq y_{ro}^{g},{\bar{y}}_{b}\geq y_{ao}^{b}\\ \;\;\;\;\;\;\;\;\;\;\;\;\;\;\;\;\;\;\;\;\;\;\;\;\;\;\;\;\;\;\;\;\;\;\;\;\;\;\;\;\;\;\;\;\;\;\;\sum_{j=1,j\neq 0}^{n}\;\lambda_{j}=1,y^{g}\geq 0,y^{b}\geq 0,\lambda \geq 0\\ \;\;\;\;\;\;\;\;\;i=1,2,\cdots ,m,a=1,2,\cdots ,q;j=1,2,\cdots ,n(j\neq 0) \end{array}\right.\;$$ (2)

In this formulation, *m* represents the categories of input factors, *n* denotes the number of provincial units, *p* signifies the efficiency value of decision-making units, *y*_*g*_ and *y*_*b*_ respectively represent the grain yield and agricultural carbon emissions per unit area, *λ* constitutes the weight vector, and *s*_*1*_ and *s*_*2*_ are the slack variables corresponding to grain yield and agricultural carbon emissions, respectively.

For calculating agricultural carbon emissions that function as an undesirable output in the Super-SBM model, this study employs the IPCC methodology due to its practical applicability and accessibility of required data. Currently, the predominant methodologies for quantifying agricultural carbon emissions include life cycle assessment, input-output analysis, and the IPCC approach. The carbon emission calculation is expressed as:


$$E=\sum E_{i}=\sum \;T_{i}\times \delta_{i}$$ (3)

where E represents the total agricultural carbon emissions, *T*_*i*_ is the carbon emission from the ith source, and *δ*_*i*_ is the emission coefficient for the *i*^*th*^ source. This study categorizes carbon sources into six types: fertilizers, pesticides, agricultural film, diesel used in agriculture, tillage, and agricultural irrigation. A detailed account of the carbon emission sources and their coefficients for all agricultural sectors is provided in Table 2.

**Table 2:** Agricultural carbon emission sources and coefficients.

Carbon-emission source	Coefficient
Fertilizer	0.8956 kg·kg^−1^
Chemical pesticide	4.9341 kg·kg^−1^
Agricultural film	5.18 kg·kg^−1^
Diesel oil used in agriculture	0.5927 kg·kg^−1^
Tillage	312.6 kg·km^−2^
Irrigation	20.476 kg·km^−2^

Drawing upon previous research [44], this study delineates the specific indicators utilized within the Super-SBM model, as summarized in Table 3. Input indicators encompass cultivated land, labor, fertilizers, pesticides, agricultural film, irrigation, diesel, and agricultural machinery. The model’s desired output is quantified as grain yield, whereas the undesirable output is captured through agricultural carbon emissions.

**Table 3:** Input and output variables for the measure of ACEE.

Element	Variable	Variable explanation	Unit
Input	Cultivated land input	Sown area of crops	10^3^hectares
	Labor input	Population employed in primary industry	10^4^persons
	Fertilizer input	Consumption of fertilizers	Tons
	Pesticide input	Consumption of pesticides	Tons
	Agricultural film input	Consumption of agricultural film	Tons
	Irrigation input	Effective irrigation area	10^3^ hectares
	Diesel input	Consumption of diesels	Tons
	Agricultural machinery input	Total power of agricultural machinery	kW
Desirable output	Output of grain	Crop grain production	Tons
Undesired output	Carbon emissions	Total carbon emissions from 6 types of carbon emission sources	Tons

#### 3.3.2. Food security assessment.

Drawing upon the FAO’s definition of food security and incorporating the distinct national circumstances of China, this research establishes a comprehensive food security assessment index system (see Table 4). This framework encompasses four pivotal dimensions: food supply security, food access security, food production stability, and food production sustainability [45]. The study employs the entropy-weighted Technique for Order Preference by Similarity to Ideal Solution (TOPSIS) method for a quantitative evaluation of food security levels, allocating weights to various indicators. To ensure temporal comparability of food security scores throughout the study period (2007–2021), we recalculated the entropy weights annually rather than using fixed weights. This approach accounts for the dynamic nature of food security systems and allows for more accurate representation of year-specific conditions and priorities. The entropy weight method determines indicator weights based on the degree of variation in the data, with greater weight assigned to indicators showing more significant differences among evaluation objects. The TOPSIS method then evaluates alternatives based on their geometric distance from both the positive ideal and the negative ideal solutions. Detailed methodological procedures and mathematical formulations are documented in Supplementary Material S1.

**Table 4:** Assessment indicator system of FS.

Overall index	First-level indicators	Secondary-level indicators	Unit	Indicators sign
Food security	Food supply security	Grain sowing area	hm^2^	+
		Per capita grain ownership	kg/people	+
	Food access security	Road network density	km·km^-2^	+
		Rural per capita disposable income	yuan/people	+
		Rural Engel’s coefficient	%	_
	Food production stability	Grain yield volatility	%	_
		Grain price volatility	%	_
		Crop disaster ratio	%	_
	Food production sustainability	Fertilizer application quantity	kg/hm^2^	_
		Pesticide application quantity	kg/hm^2^	_

#### 3.3.3. Coupling coordination degree model.

The CCD model is employed to quantitatively assess the synergistic benefits and the alignment of their combined impact with the overall effect arising from the coupling between ACEE and FS. The mathematical formulation of the model is delineated as follows:


$$C=2\frac{\sqrt{U_{1}U_{2}}}{U_{1}+U_{2}}\;$$ (4)

$$D=\sqrt{CT},T=\alpha U_{1}+\beta U_{2}$$ (5)

where *C* represents the degree of coupling between ACEE and FS, ranging from 0 to 1. *U*_*1*_ and *U*_*2*_ denote the scores of the ACEE and FS subsystems, respectively. *T* represents the comprehensive coordination index of ACEE and FS, where *α* and *β* are weighting parameters, both assigned a value of 0.5. *D* indicates the coupling coordination degree, also ranging from 0 to 1, with larger values indicating higher degrees of coordination. The classification framework for CCD is detailed in Table 5:

**Table 5:** Classification of CCD.

Coupling Coordination Degree	Coupling Coordination Stage
0.000 ≤ D ≤ 0.099	Extremely Disorder
0.100 ≤ D ≤ 0.199	Severe Disorder
0.200 ≤ D ≤ 0.299	Moderate Disorder
0.300 ≤ D ≤ 0.399	Mild Disorder
0.400 ≤ D ≤ 0.499	Imminent Disorder
0.500 ≤ D ≤ 0.599	Barely Coordinated
0.600 ≤ D ≤ 0.699	Primary Coordination
0.700 ≤ D ≤ 0.799	Intermediate Coordination
0.800 ≤ D ≤ 0.899	Good Coordination
0.900 ≤ D ≤ 1.000	High-quality Coordination

Furthermore, this research utilizes the ratio *U*_*1*_/*U*_*2*_ to elucidate the developmental disparities between the subsystems. A ratio exceeding 1 indicates that ACEE development surpasses the FS system, underscoring the imperative to bolster FS to strengthen the coupling coordination degree. Conversely, a ratio below 1 signifies that ACEE lags behind the FS system, necessitating different strategic interventions.

#### 3.3.4. Spatial-temporal analysis method.

(1) Global spatial autocorrelation (GA)

Global spatial autocorrelation, which quantifies the degree of correlation among variables across the entire study area, is measured using the global Moran’s I. The mathematical expression is formulated as follows:


$$Global\;Moran^{\prime }I=\frac{n\sum_{i=1}^{n}\;\sum_{j=1}^{n}\;w_{ij}\left(x_{i}-\bar{x}\right)\left(x_{j}-\bar{x}\right)}{\sum_{i=1}^{n}\;\sum_{j=1}^{n}\;w_{ij}\sum_{i=1}^{n}\;{\left(x_{i}-\bar{x}\right)}^{2}}$$ (6)

where *n* represents the number of samples; *x*_*i*_ and *x*_*j*_ indicate the coupling coordination degrees of regions *i* and *j*; $\bar{x}$ represents the mean value of all measurements; *w*_*ij*_ represents the spatial weight matrix; and *I* represents the Moran’s I index, ranging from −1–1. A value greater than 0 indicates positive spatial correlation, less than 0 indicates negative spatial correlation, and equal to 0 implies no spatial correlation. The significance of Moran’s I is evaluated through the standardized statistic *Z*:


$$Z=\frac{I-E(I)}{\sqrt{VAR(I)}}$$ (7)

where *E*(*I*) represents the expected value of Moran’s I, and *VAR* represents its variance.

(2) Local indicators of spatial association (LISA) clustering

In this study, local indicators of spatial association are employed to elucidate the spatial clustering patterns of coupling coordination degrees at the provincial level. Specifically, the local Moran’s I is applied to identify local clustering effects, categorizing the spatial-temporal distributions into four distinct typologies: high-high (H-H), low-low (L-L), high-low (H-L), and low-high (L-H) clusters. The mathematical formulation is presented below, with variable definitions consistent with those in Equation (6):


$$Local\;Moran^{\prime }I=(\frac{x_{i}-\;\bar{x}}{\text{n}}){\sum {\mathrm{\backslash nolimits}}_{i=1}^{n}w}_{ij}\left(x_{j}-\bar{x}\right)$$ (8)

(3) Markov Chain

The Markov chain methodology elucidates the state transition dynamics and quantifies the corresponding transition probabilities [46], facilitating the examination of the evolutionary characteristics of CCD. Based on established classification criteria for CCD, the data are stratified into five distinct categories (k = 5). The evolutionary dynamics of geographical phenomena are conceptualized as a Markov process by quantifying the probability distributions and variations across categorical states. The comprehensive state transition process is represented through a Markov transition probability matrix, with *M*_*ij*_ denoting the transition probability value. This framework employs the 1 × k state probability vector *E*_*t*_=[*E*_*1,t*_,*E*_*2,t*_,…,*E*_*k,t*_] to represent a specific probability distribution at time *t*. The dynamic evolution is mathematically expressed as follows:


$$M_{ij}=\;n_{ij}/n_{i}$$ (9)

where *M*_*ij*_ represents the probability of transition from type-*i* spatial unit to type-*j* spatial unit between time *t* and *t* + 1, *n*_*ij*_ represents the total number of spatial units transitioning from type-*i* to type-*j* during this temporal interval, and *n*_*i*_ represents the total number of type-*i* spatial units observed throughout the study period.

The spatial Markov chain integrates the concept of spatial lag, thereby addressing the limitation of conventional Markov chains that neglect spatial interactions. A transition probability matrix is constructed under various spatial lag conditions by incorporating a spatial weight matrix to calculate the spatial lag of adjacent regions. The transition probability matrix of the spatial Markov chain can be decomposed into *k* conditional transition matrices of dimension *k* × *k*. The term *m*_*ij*_ (*k*) denotes the transition probability of a spatial unit at time *t* transitioning to type-*j* at time *t* + 1, conditional upon spatial lag type-*k*. The spatial lag classification is determined by the spatial lag value, which represents the local spatially weighted average of attribute values in neighboring areas. The mathematical formulation is expressed as:


$$Lag\;=Y_{i}\;W_{ij}$$ (10)

where *Y*_*i*_ represents the attribute value of the spatial unit, while *W*_*ij*_ represents the adjacency weight matrix or the spatial relationship matrix between the focal unit and its neighboring regions.

#### 3.3.5. Combination forecasting model.

The combination forecasting model integrates the GM (1,1), ARIMA, and LSTM methodologies to predict the future trends of CCD levels between ACEE and FS from 2022 to 2030. This approach synthesizes the strengths of diverse forecasting paradigms to enhance predictive accuracy [47]. The methodological framework is delineated as follows:

Step 1: Constructing the combination forecasting model


$${\hat{f}}_{t}=w_{1,t}\cdot {\hat{f}}_{1,t}+w_{2,t}\cdot {\hat{f}}_{2,t}+\cdots +w_{(m-1),t}\cdot {\hat{f}}_{m-1,t}+w_{m,t}\cdot {\hat{f}}_{m,t}$$ (11)

$$\begin{array}{c} \;\sum {\mathrm{\backslash nolimits}}_{i=1}^{m}\;w_{i,t}=1,w_{i,t}\geq 0,t=1,2,\cdots ,n \end{array}$$ (12)

where ${\hat{f}}_{t}\;$and ${\hat{f}}_{1,t}$ represent the combination forecast and individual model forecasts for year *t*, respectively. *w*_*i*,*t*_ represents the weight of the *i*^*th*^ individual forecasting model at time *t*.

Step 2: Residual calculation


$$e_{i,t}=y_{t}-{\hat{f}}_{i,t}$$ (13)

$$\begin{array}{c} e_{t}=y_{t}-{\hat{f}}_{t}=\sum {\mathrm{\backslash nolimits}}_{i=1}^{m}\;w_{i}\cdot e_{i,t} \end{array}$$ (14)

where *y*_*t*_ represents the actual value at time *t*. *e*_*i*,*t*_ (or *e*_*t*_) represents the residual errors between the actual and forecast values of the *i*^*th*^ individual model (or combination prediction model) for year *t*^*th*^.

Step 3: Weight determination


$$\left\{ \begin{array}{c} \;\;\;\;\;\;\;\;\;\;\;\;\;\;\;\;\;\;\;\;\;\;\;\;\;\;\;\;\;\;\;\;\;\min J_{t}-\left|e_{t}\right|=\sum_{i=1}^{m}\;w_{i,t}\cdot e_{i,t}\\ \;\;\text{\textasciitilde{}s.t.\textasciitilde{}}\sum_{i=1}^{m}\;w_{i,t}=1,w_{i,t}\geq 0,t=1,2,\cdots ,n \end{array}\right.\;$$ (15)

Two algorithmic solutions are proposed for the above model. Firstly, when the residual errors *e*_*i*,*t*_ of all individual models are consistently greater or less than 0, and the *p*^*th*^ model demonstrates the smallest absolute residual error, then its weight *w*_*p*,*t*_ is 1, while those of other models are 0. Secondly, if individual model residual errors *e*_*i*,*t*_ are both greater and less than 0, the weight of an individual forecasting model can be measured by Equation (15). Moreover, Equation (16) can be used to calculate the modeling weight of the forecast time period (t = b + 1, b + 2,..., n).


$$\omega_{i,t}=\frac{e_{i,t}^{-1}}{\sum_{i-1}^{k}\;e_{i,t}^{-1},\;\forall i=1,2,\ldots ,k}$$ (16)

$$\begin{array}{c} w_{i,(b+1)}=\frac{1}{b}\sum {\mathrm{\backslash nolimits}}_{t=1}^{b}\;w_{i,},w_{i,(b+2)}=\frac{1}{b}\cdot \sum {\mathrm{\backslash nolimits}}_{t=2}^{b+1}\;w_{i,s},\ldots ,w_{i,n}=\frac{1}{b}\sum {\mathrm{\backslash nolimits}}_{t=n}^{n-1}\;w_{i,s} \end{array}$$ (17)

Details of individual forecasting models can be seen in the S2 of the Supplementary Material. The mean absolute error (MAE), root mean square error (RMSE), and mean absolute percentage error (MAPE) are used to evaluate the predictive performance of the models. The formulas are as follows:


$$MAE=\frac{1}{n}\sum_{t=1}^{n}\;\left|y_{t}-{\hat{f}}_{i,t}\right|$$

$$\begin{array}{c} RMSE=\sqrt{\frac{1}{n}\sum {\mathrm{\backslash nolimits}}_{t=1}^{n}\;{\left(y_{t}-{\hat{f}}_{i,t}\right)}^{2}} \end{array}$$ (18)

$$\begin{array}{c} \text{MAPE =}\frac{100\%}{n}\sum {\mathrm{\backslash nolimits}}_{t=1}^{n}\;\left|\frac{y_{t}-{\hat{f}}_{i,t}}{y_{t}}\right| \end{array}$$

## 4. Results

### 4.1. Spatial-temporal distribution of ACEE and FS

It is important to note that the ACEE and FS indices presented in this study are relative scores rather than absolute measures. Higher ACEE values indicate greater efficiency in agricultural production with lower carbon emissions, while higher FS values represent better performance across food supply, access, stability, and sustainability dimensions relative to other provinces. These comparative indices are useful for identifying regional disparities, tracking temporal trends, and formulating targeted policies.

#### 4.1.1. Spatial-temporal distribution of ACEE.

**Fig 2:**
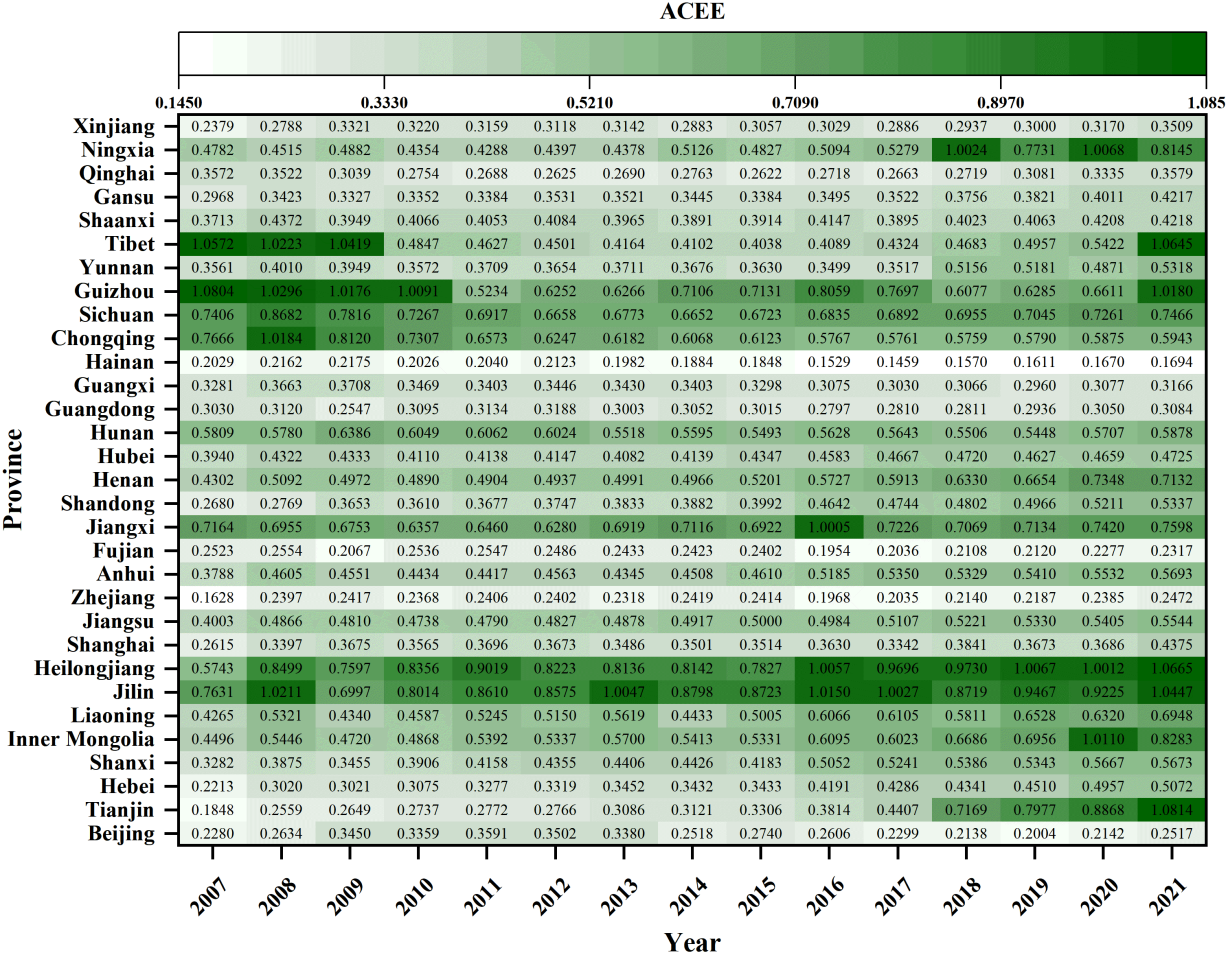
Spatial-temporal distribution of ACEE in Chinese provinces from 2007 to 2021.

This research reveals significant potential for enhancing ACEE, with the efficiency values of the majority of provinces not exceeding 0.6 (see Fig 2). Temporal trend analysis indicates a fluctuating yet overall upward trajectory in ACEE across most provinces, with notable exceptions including Guizhou, Chongqing, Hainan, Guangxi, and Fujian. Spatial analysis identifies the northeastern and central regions as exhibiting higher ACEE, attributable to favorable geographical conditions and advanced technological adoption (see Supplementary Material Fig. S1). In contrast, the western region demonstrates considerable heterogeneity in efficiency, with areas such as Tibet and Guizhou performing well, while Qinghai and Xinjiang lag behind. The eastern region, initially registering lower efficiency, exhibits progressive improvement over time. Overall, regional disparities in ACEE are gradually diminishing, with high-efficiency areas expanding westward into agricultural-based regions [9]. This trend coincides with strategic initiatives for agricultural restructuring and increased financial investments as part of the western development campaign.

#### 4.1.2. Spatial-temporal distribution of FS.

**Fig 3:**
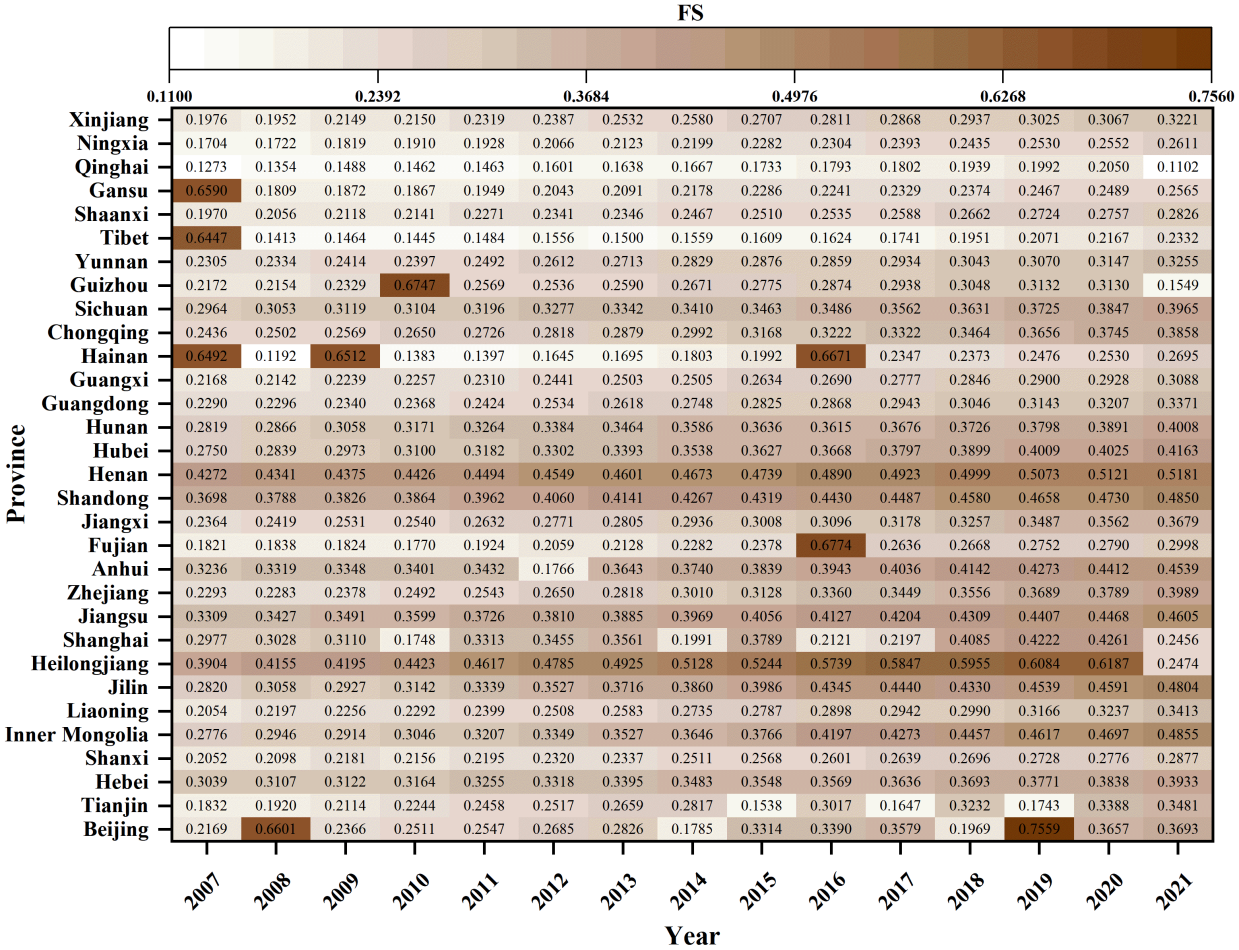
Spatial-temporal distribution of FS in Chinese provinces from 2007 to 2021.

An analysis of the temporal and spatial distribution of FS reveals that most provinces exhibit FS levels below 0.5 from 2007 to 2021, indicating a considerable potential for improvement (see Fig 3). Excluding Heilongjiang, Shanghai, Hainan, Guizhou, Tibet, Gansu, and Qinghai, a general upward trend in FS is observed, primarily attributed to government investments in agricultural infrastructure and technological advancements. Spatially, FS exhibits significant heterogeneity [48], with northeastern and central regions, which are major grain-producing areas, achieving higher FS levels due to advantageous natural conditions and robust governmental support for agricultural infrastructure and innovation (see Supplementary Material Fig. S2). In contrast, the western region, constrained by both natural and economic limitations, exhibits relatively lower FS levels, which aligns with the findings of previous research [49]. To address these spatial disparities in FS, strategies such as the “north-to-south grain transfer” and “central-to-west grain transfer” initiatives warrant consideration for enhancing regional and national food security.

### 4.2. Spatial-temporal distribution of CCD

#### 4.2.1. The temporal distribution of CCD.

**Fig 4:**
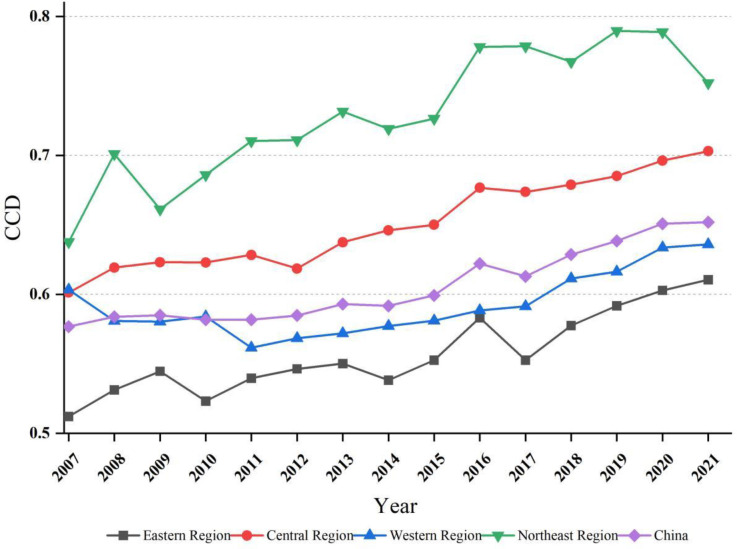
CCD in China and four major regions from 2007 to 2021.

Temporal analysis from 2007 to 2021 shows a general upward trend in the CCD between ACEE and FS in China, evolving from “barely coordinated” to “primary coordination” (see Fig 4). This trend reflects the government’s commitment to integrating environmental protection with food security imperatives. Concurrently, the period has been marked by notable fluctuations in the ACEE (U_1_) to FS (U_2_) ratio, underscoring the challenges of achieving balanced development between these entities (see Supplementary Material Fig. S3). Addressing these challenges, future efforts should prioritize strengthening agricultural supply-side reforms and enhancing the capacity and quality of agricultural output.

#### 4.2.2. The spatial distribution of CCD.

**Fig 5:**
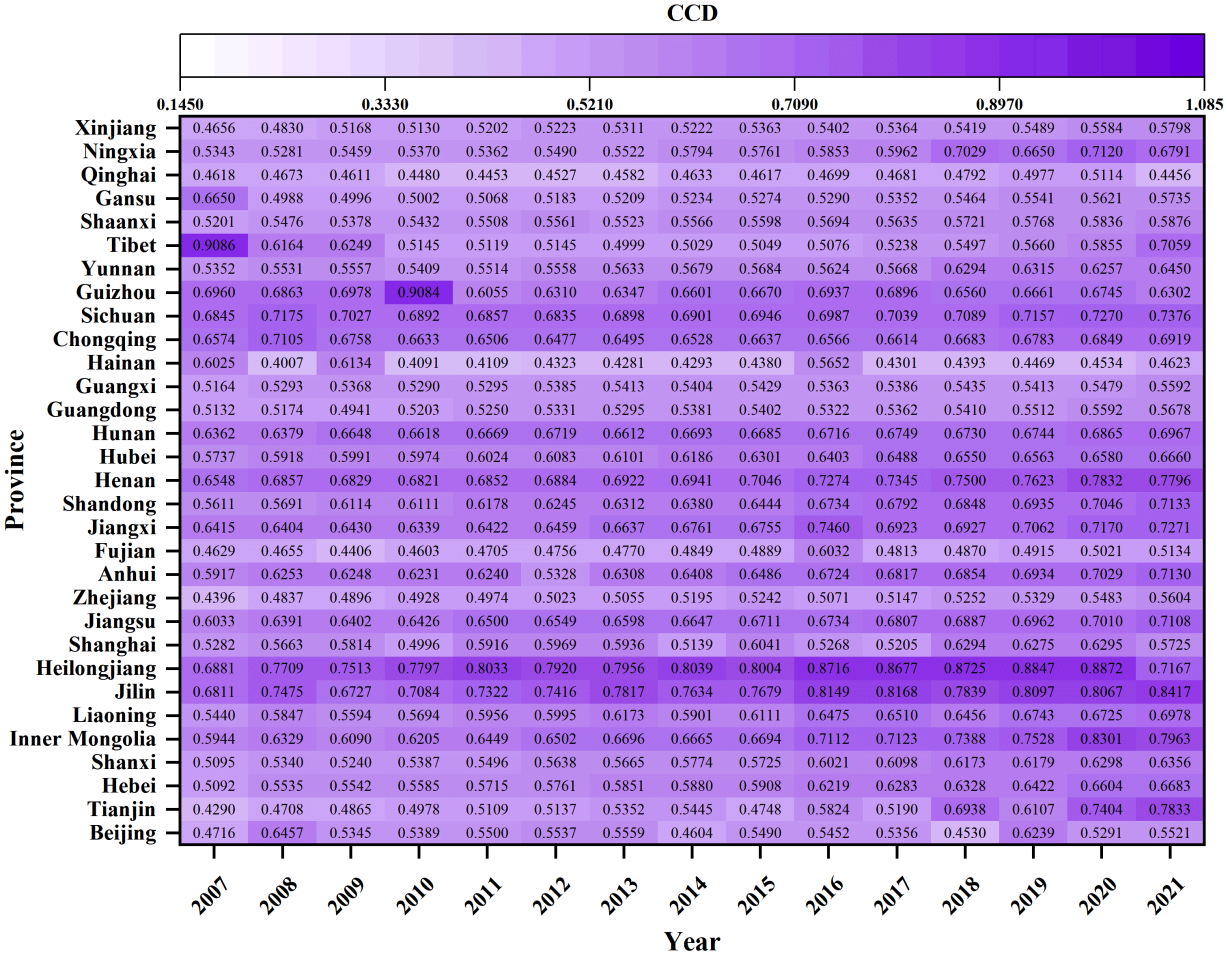
Spatial-temporal distribution of CCD in Chinese provinces in selected years.

Spatial analysis from 2007 to 2021 reveals significant variations in the CCD across Chinese provinces (see Fig 5). The levels of CCD in the northeastern and central regions are generally higher than those in the western and eastern regions. Provinces in the northeastern and central regions, such as Heilongjiang, Jilin, Liaoning, Henan, Jiangxi, and Anhui, have achieved levels of “good coordination” and “intermediate coordination”, significantly surpassing the national average. In contrast, many provinces in the west and east, especially Qinghai and Hainan, exhibit lower coordination levels, indicating potential for improvement. Notably, the evolution of CCD levels varies across regions. The northeastern and central areas have seen continuous improvement, especially in Liaoning, Jilin, Shanxi, and Henan (see Supplementary Material Fig. S4-S5). Meanwhile, the western region has experienced a period of decline before rebounding, with Inner Mongolia and Ningxia contributing significantly to the recovery (see Supplementary Material Fig. S6). The eastern region, notably Tianjin, experienced a significant transition from “barely coordinated” to “primary coordination” (see Supplementary Material Fig. S7). Despite the overall upward trend in CCD across most provinces, the necessity for region-specific strategies emerges as a critical imperative, particularly for improving CCD levels in the western and eastern provinces.

### 4.3. Spatial correlation characteristics and evolutionary probability of CCD

#### 4.3.1. Global spatial autocorrelation analysis.

**Fig 6:**
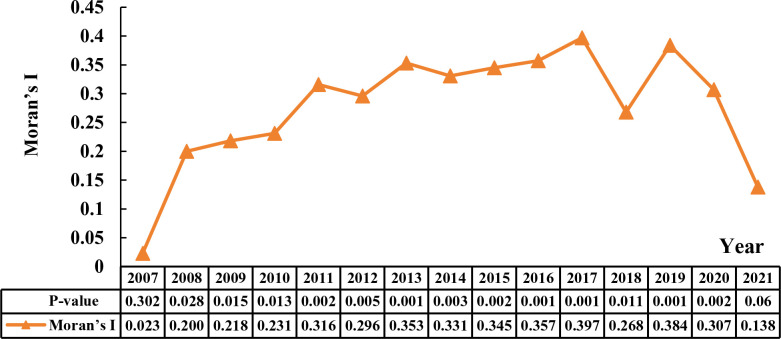
The trend of global Moran’s I index from 2007 to 2021.

Employing ArcGIS and GeoDa software, this study utilizes the global Moran’s I to assess the spatial correlation of CCD among 31 provinces in China (see Fig 6). The findings reveal that the Moran’s I values throughout the study period are uniformly positive, with the majority attaining statistical significance at the 5% level. This result rejects the null hypothesis of no spatial autocorrelation within the CCD distribution. Subsequent analysis of the temporal trends of Moran’s I values from 2007 to 2021 uncovers an initial increase, signifying a strengthening of spatial positive correlation and a pronounced clustering of regions with both high-high and low-low CCD levels. This phase indicates increasing spatial disparities between regions with high and low CCD. The subsequent decline in the Moran’s I reflects the positive influence of state policy interventions and targeted agricultural resource allocations towards regions with lower CCD. Such measures have contributed to mitigating the disparities between high and low CCD areas, thereby underscoring the efficacy of national policies in promoting coordinated regional agricultural development.

#### 4.3.2. LISA clustering analysis.

The LISA clustering analysis of the CCD across 31 provinces in China from 2007 to 2021 was conducted using ArcGIS software, as shown in Table 6. This analysis aimed to explore the local spatial characteristics of CCD. Data from selected years (2007, 2012, 2017, and 2021) underwent visual examination to identify high-high, low-low, low-high, and high-low spatial adjacencies. The findings revealed pronounced regional clustering patterns in carbon-food coupling coordination. Notably, provinces in the northeast (Heilongjiang, Jilin, and Liaoning) exhibited significant high-high clusters, demonstrating their success in integrating low-carbon agricultural practices with food production, as they are China’s principal grain-producing areas. Over time, Shandong has also emerged as a high-high cluster. The reduction in the low-high clustering effect in Qinghai, Yunnan, and Guangdong indicates a positive impact from the coordinated development of adjacent regions. Conversely, Tibet, classified as a high-low cluster, encounters challenges due to the lower coordination levels of neighboring provinces, underscoring the necessity for enhanced technological support and regional cooperation to elevate the overall CCD level.

**Table 6:** LISA clustering distribution of CCD in Chinese provinces in selected years.

	2007	2012	2017	2021
High-High Cluster	Heilongjiang, Sichuan	Heilongjiang, Jilin	Heilongjiang, Jilin, Liaoning	Heilongjiang, Jilin, Liaoning, Shandong
High-Low Outlier	—	—	—	Tibet
Low-High Outlier	Qinghai, Yunnan	Guangdong	Guangdong	Shaanxi
Low-Low Cluster	—	Xinjiang	Xinjiang	—

#### 4.3.3. Markov chain analysis.

This study employs the Markov chain methodology to analyze the dynamic evolutionary probability of CCD across different provinces. This approach assumes stationary transition probabilities over the study period (2007–2021), implying that the probability of a province transitioning from one CCD category to another remains relatively constant over time. While this assumption is a simplification of complex economic systems, it is justified in our context by the relatively short time span and the overall stability of China’s agricultural and environmental policy framework during this period.

Table 7 presents the traditional Markov transition probability matrix for the CCD. The analysis reveals several key insights: First, most provinces are categorized within the “barely coordinated” and “primary coordination” levels, suggesting considerable potential for enhancing CCD. Second, the values on the diagonal are the largest, indicating that provinces are more likely to maintain their current CCD level than to transition, with at least a 71.4% probability of retaining their status. This finding reflects the considerable inertia in provincial CCD systems, which is likely attributable to deeply embedded socio-economic structures, policy continuity, and regional development path dependencies that collectively resist rapid change. Furthermore, the data reveal a persistent stratification pattern where provinces tend to remain within their initial CCD category, a phenomenon analogous to what economic literature terms “poverty traps” or “development clubs”. This pattern suggests structural barriers to mobility between different development stages, where provinces with initially higher coordination levels maintain advantages through accumulated technological capacity, institutional quality, and resource allocation efficiency. Rather than representing deliberate exclusion, this stratification reflects self-reinforcing advantages that higher-performing provinces have established through prior development efforts.

**Table 7:** Traditional Markov transition probability matrix for CCD.

t/t + 1	Observations	Ⅰ	Ⅱ	Ⅲ	Ⅳ
Ⅰ	56	**0.714**	0.214	0.071	0.000
Ⅱ	166	0.048	**0.861**	0.078	0.012
Ⅲ	158	0.025	0.044	**0.829**	0.101
Ⅳ	54	0.000	0.000	0.148	**0.852**

Note: I-Imminent Disorder, Ⅱ-Barely Coordinated, Ⅲ-Primary Coordination, Ⅳ-Intermediate Coordination.

The transitions in CCD types predominantly occur adjacent to the diagonal of the matrix, indicating incremental rather than transformative change. This finding has profound implications for policy formulation, suggesting that interventions aiming for dramatic improvements in CCD may be less effective than sustained, incremental approaches that gradually overcome systemic barriers. The high retention probability (85.2%) in the highest CCD category further suggests that once provinces achieve “intermediate coordination,” they develop self-reinforcing mechanisms that maintain their advantageous position, potentially through virtuous cycles of technological innovation, institutional learning, and resource optimization.

Leveraging the geographical interconnections among Chinese provinces, this study extends the traditional Markov chain model by integrating spatial dimensions through the spatial Markov approach, which relaxes the assumption of spatial independence among observations. This method calculates conditional transition probabilities based on neighboring provinces’ CCD status, as presented in Table 8. The spatial Markov analysis acknowledges that transition probabilities may vary based on spatial context, providing a more realistic representation of regional economic dynamics.

**Table 8:** Spatial Markov transition probability matrix for CCD.

Space Lag	t/t + 1	Observations	Ⅰ	Ⅱ	Ⅲ	Ⅳ
Ⅰ	Ⅰ	2	**0.500**	0.000	0.500	0.000
	Ⅱ	6	0.000	**1.000**	0.000	0.000
	Ⅲ	1	1.000	0.000	**0.000**	0.000
	Ⅳ	0	0.000	0.000	0.000	**0.000**
Ⅱ	Ⅰ	51	**0.725**	0.235	0.039	0.000
	Ⅱ	91	0.077	**0.868**	0.055	0.000
	Ⅲ	48	0.063	0.063	**0.813**	0.063
	Ⅳ	8	0.000	0.000	0.250	**0.750**
Ⅲ	Ⅰ	3	**0.667**	0.000	0.333	0.000
	Ⅱ	67	0.015	**0.836**	0.119	0.030
	Ⅲ	103	0.000	0.039	**0.835**	0.126
	Ⅳ	32	0.000	0.000	0.188	**0.813**
Ⅳ	Ⅰ	0	**0.000**	0.000	0.000	0.000
	Ⅱ	2	0.000	**1.000**	0.000	0.000
	Ⅲ	6	0.000	0.000	**1.000**	0.000
	Ⅳ	14	0.000	0.000	0.000	**1.000**

This spatial analysis reveals critical insights that significantly refine our understanding of regional CCD dynamics. Most notably, Table 8 demonstrates that a province’s CCD trajectory is substantially influenced by its neighbors’ status, creating spatial dependency structures that transcend provincial boundaries. Provinces surrounded by high-level neighbors (Type IV spatial lag) exhibit perfect state persistence (100% probability of maintaining status), revealing a stabilizing effect that suggests policy diffusion, knowledge spillovers, and resource-sharing networks among high-performing clusters. Conversely, provinces with Type III spatial lag demonstrate enhanced upward mobility potential, with transition probabilities from lower to higher states exceeding those in the traditional model. This provides evidence for positive spatial externalities where intermediate-high performing regions exert beneficial influence on surrounding areas. The practical significance of this finding is substantial: strategic placement of policy interventions in carefully selected provinces could catalyze improvements across entire regions through these spatial mechanisms.

The spatial Markov findings further indicate that geographical context can either amplify or mitigate the stratification effect observed in the traditional model. The specific transition probabilities in Table 8 suggest that provinces in low-high clusters (like Qinghai, Yunnan, Guangdong, and Shaanxi) represent strategic leverage points where targeted interventions could yield disproportionate benefits by exploiting positive neighborhood effects. Conversely, provinces in high-low clusters (particularly Tibet) face exceptional challenges due to negative spatial externalities, requiring customized policy approaches that account for their unique geographical circumstances.

### 4.4. Combination forecasting of CCD

The combination forecasting model is employed to predict the CCD between ACEE and FS from 2022 to 2030. This model integrates the GM(1,1), ARIMA, and LSTM methodologies to enhance predictive accuracy. The GM(1,1) model is characterized by its minimal data requirements and high fitting accuracy for small samples. The ARIMA model is known for its adaptability to diverse data characteristics, making it suitable for non-stationary time series analysis. The LSTM model, with its self-learning capabilities, excels in addressing complex nonlinear challenges. It is important to note that our predictions operate under several key assumptions: (1) relative stability in China’s agricultural and environmental policy framework; (2) continuation of current technological and socioeconomic development trends; and (3) absence of extreme events that might significantly disrupt established patterns. Should these conditions change substantially, actual outcomes may differ from our projections.

As shown in Supplementary Material Table S1, the individual models achieve high predictive accuracy, evidenced by near-zero MAE, RMSE, and MAPE. Among the various forecasting methodologies, the combination forecasting model exhibits the highest precision in predicting the CCD across various provinces (see Supplementary Material Table S2). The weight distribution of each model within the combination forecasting approach is comprehensively detailed in Supplementary Material Tables S3 and S4, reflecting the optimal integration of the three forecasting approaches based on historical data patterns.

**Fig 7:**
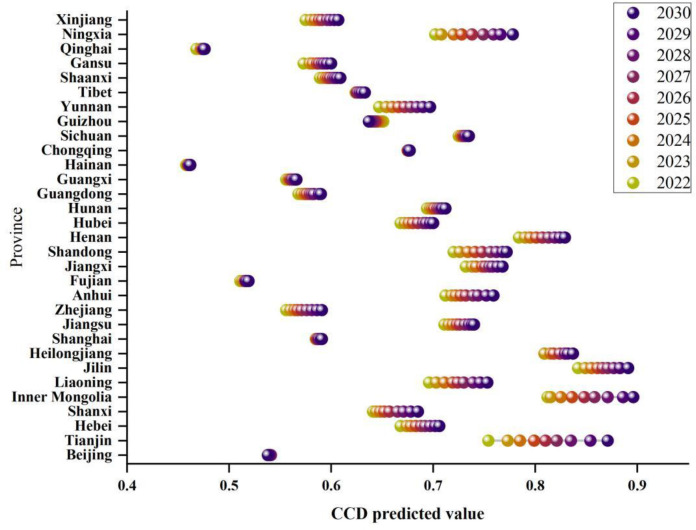
The prediction of CCD in Chinese provinces from 2022 to 2030.

The prediction results reveal a year-on-year increasing trend in CCD across the four major regions from 2022 to 2030, with significant inter-regional differences, as shown in Supplementary Material Fig. S5. By 2030, the northeast region is expected to exhibit the highest CCD value at 0.827, followed by the central region at 0.742, the national average at 0.685, the western region at 0.659, and the eastern region at 0.638. The growth rates for these regions are anticipated at 5.72%, 5.26%, 4.84%, 4.11%, and 5.12%, respectively. This prediction indicates that the western and eastern regions have relatively lower CCD levels, impacting the national average, yet the eastern region exhibits potential for growth. From a provincial perspective, most provinces are expected to experience an upward trend in CCD levels, although the rates of growth vary. Tianjin and Inner Mongolia are expected to experience significant increases, while Qinghai, Sichuan, Hainan, Fujian, and Shanghai are expected to show modest rises. Chongqing’s CCD level is likely to remain stable, with slight declines anticipated in Beijing and Guizhou (see Fig 7). By 2030, excluding Qinghai and Hainan, all other provinces are expected to reach or surpass the “barely coordinated” level.

The provinces exhibiting extreme results warrant particular analysis. The significant increases projected for Tianjin and Inner Mongolia likely stem from their ongoing structural transformations in agricultural practices and strong policy support for low-carbon development. In Tianjin, the integration of modern agricultural technology with environmental protection initiatives has accelerated in recent years, while Inner Mongolia has made substantial investments in sustainable grazing practices and efficient irrigation systems. Conversely, the persistent low CCD levels predicted for Qinghai and Hainan can be attributed to their distinct geographical and ecological constraints. Qinghai faces challenges from its high-altitude plateau environment and limited arable land, which restrict agricultural development options. Hainan’s tropical island ecosystem presents unique vulnerabilities to climate change impacts, particularly affecting agricultural stability. The slight declines projected for Beijing and Guizhou reflect different challenges: Beijing’s continuing urbanization pressure on agricultural land and Guizhou’s mountainous terrain limiting large-scale agricultural modernization. Consequently, future strategies should focus particularly on these regions with lower CCD levels (Qinghai, Hainan) or those exhibiting declining trends (Beijing, Guizhou), with policies tailored to their specific constraints and development potentials.

## 5. Conclusion and policy implications

### 5.1. Conclusion

Climate change and food security represent critical challenges to agricultural sustainable development goals, especially in a country as large and populous as China. Understanding the interplay between ACEE and FS through the perspective of CCD is crucial for guiding the transition towards low-carbon agriculture and sustainable development across Chinese provinces. This study assesses ACEE and FS across 31 provinces in China from 2007 to 2021, employing the CCD model to evaluate the coupling relationship between ACEE and FS. The GA, LISA clustering, Markov chain, and combination forecasting models are used to explore the spatial-temporal characteristics and evolutionary probabilities. By analyzing the ACEE-FS CCD and regional disparities from both temporal and spatial perspectives, this research reveals:

(1) Throughout the study period, China’s ACEE and FS exhibited a fluctuating yet overall upward trend, with high spatial disparities observed. The northeastern and central regions demonstrated consistently higher ACEE values, in contrast to the notable spatial variability in the western region and a progressively increasing trend in the eastern coastal provinces. Concurrently, regions with high FS values were primarily located in the major grain-producing areas of the northeast and central regions, contrasting with the western region’s lower FS levels. This pattern underscores the interrelation between geographical location, agricultural practices, and food security outcomes, highlighting regional strengths and identifying areas requiring focused development efforts to enhance ACEE and FS.
(2) Throughout the study period, the CCD between ACEE and FS showed a progressive increase, evolving from “barely coordinated” to “primary coordination”. This advancement underscores the growing integration of ACEE and FS across China. However, regional disparities were pronounced, with the northeastern and central regions achieving higher levels of coordination than their western and eastern counterparts. Notably, an imbalance between ACEE and FS became evident, particularly with FS trailing behind ACEE. This observation highlights an urgent need for targeted strategies aimed at bolstering FS, thereby maximizing the benefits of a more harmoniously coordinated development between ACEE and FS.
(3) This research uncovers distinct temporal and spatial dynamics in the evolution of CCD between ACEE and FS. Through global spatial autocorrelation analysis, a pronounced positive correlation was identified, indicating significant spatial clustering of CCD. This pattern was further substantiated by LISA clustering analysis, which revealed persistent high-high clusters in the northeast, alongside emerging clusters in regions like Shandong. These findings suggest effective integration of carbon reduction and food security strategies. Moreover, Markov chain analysis illuminated the dynamic evolutionary probability of CCD, revealing a tendency towards regional “club convergence”, underscoring the impact of geographical proximity on CCD stability. This finding emphasizes the importance of targeted government interventions in regions exhibiting low-low clusters, such as Xinjiang, and high-low clusters, like Tibet, to counteract potential stagnation and promote balanced development.
(4) The combination forecasting model was used to predict the CCD across 31 provinces in China. The analysis indicates that the northeastern region is predicted to achieve the highest CCD level, followed by the central region, while the western and eastern regions are expected to exhibit lower levels. From a provincial perspective, significant variations in forecasted CCD changes are observed. Tianjin and Inner Mongolia are predicted to experience the most substantial increases, whereas Beijing and Guizhou are expected to exhibit slight decreases. However, by 2030, with the exception of Qinghai and Hainan, the CCD for all provinces is anticipated to surpass the “barely coordinated” threshold. This prediction highlights the regional disparities in CCD evolution and underscores the importance of targeted interventions to enhance coordination between ACEE and FS, ensuring that all regions progress beyond “barely coordinated” levels.

### 5.2. Policy implications

In conclusion, the findings of this research contribute to advancing the coordinated development between ACEE and FS in China. Currently, many provinces are classified within the “barely coordinated” and “primary coordination” levels, indicating substantial opportunities for improvement. Drawing upon the insights gained from this study, we propose the following targeted policy recommendations:

(1) Implement a spatially targeted “coordination enhancement” strategy based on LISA clustering results

Our LISA clustering analysis revealed distinct spatial patterns in CCD distribution, with high-high clusters in northeastern provinces and various transition zones between high and low CCD regions. Rather than applying uniform policies nationally, we recommend establishing a differentiated policy framework that targets: (a) “Bridge provinces” that connect high-CCD and low-CCD clusters (such as Shaanxi, identified in our analysis as evolving from a low-high to a non-significant cluster); (b) “Consolidation zones” in northeastern provinces with established high-high clusters where maintaining coordination is critical; and (c) “Transformation priorities” in regions showing persistent low-low patterns (particularly Xinjiang as identified in our Markov analysis). This spatial targeting approach, directly informed by our research findings, would optimize resource allocation and maximize policy effectiveness.

(2) Develop dynamic adjustment mechanisms based on our Markov transition probability findings

Our Markov chain analysis revealed that provinces have at least a 71.4% probability of maintaining their current CCD status, indicating significant system inertia. Rather than conventional static policies, we recommend establishing dynamic policy mechanisms with predefined intervention thresholds based on our calculated transition probabilities. Specifically, in provinces where spatial Markov analysis indicates a probability exceeding 20% for CCD level decline (as we identified in Type II spatial lag regions), automated policy responses should be triggered. This data-driven approach would provide a more responsive policy system that addresses coordination challenges before they manifest as significant declines.

(3) Address the identified ACEE-FS imbalance with targeted capacity enhancement

Our temporal analysis uniquely identified a persistent gap between ACEE and FS development, with food security consistently lagging behind carbon emission efficiency improvements. Based on this finding, we recommend prioritizing investment in food security enhancement policies that specifically address aspects performing poorly in our multi-dimensional FS assessment. For northeastern provinces, which showed high ACEE but varying FS performance in our analysis, we recommend focusing on production stability (particularly reducing yield volatility identified in our assessment framework). For western provinces, which our analysis shows face significant food access challenges, investment in rural infrastructure and income-enhancing policies would yield the greatest CCD improvements.

(4) Implement anticipatory policies based on our predictive modeling

Our combination forecasting model predicts specific trajectories for each province through 2030, providing a unique opportunity for anticipatory policy design. For provinces our model identifies as potentially experiencing CCD decline (Beijing and Guizhou), we recommend immediate intervention focusing on their specific coordination challenges—urbanization pressure in Beijing and agricultural modernization constraints in mountainous Guizhou. For provinces predicted to see significant improvements (Tianjin and Inner Mongolia), we recommend establishing regulatory frameworks that can accommodate the predicted structural changes in their agricultural systems to maintain their positive trajectory without compromising either ACEE or FS.

### 5.3. Limitations

While this study provides comprehensive insights into the coupling coordination between agricultural carbon emission efficiency and food security in China, several limitations should be acknowledged.

First, the construction of the ACEE and FS indices relies on a selected set of indicators that, while carefully chosen based on literature and data availability, may not capture all aspects of these complex systems. The agricultural carbon emission calculations primarily focus on direct emissions from agricultural activities but may not fully account for indirect emissions from the entire agricultural supply chain. Similarly, the food security assessment, while multidimensional, cannot encompass all social and cultural factors that influence food security outcomes.

Second, our forecasting models, despite combining multiple methodologies to enhance prediction accuracy, remain constrained by the historical patterns observed in the data. These predictions assume relative stability in current development trends and policy frameworks, and may not adequately capture potential disruptions from extreme climate events, major policy shifts, or technological breakthroughs. The complex interactions between agricultural systems, climate change, economic development, and policy interventions create inherent uncertainties in long-term projections.

Third, this study focuses on provincial-level analysis, which may mask significant intra-provincial variations in both ACEE and FS. The aggregation of data at the provincial level can obscure important local disparities and challenges, particularly in geographically diverse provinces. County-level or even finer-scale analyses would provide more nuanced insights but are constrained by data availability.

Despite these limitations, this study provides valuable insights into the spatial-temporal evolution of the coordination between agricultural carbon efficiency and food security in China. Future research could address these limitations by incorporating more comprehensive indicators, developing non-stationary Markov models, employing alternative forecasting approaches that better account for potential disruptions, and conducting analyses at finer spatial scales as data becomes available.

## Supporting information

S1 File Supplementary Material (DOCX)
