# Supplementary material for: Coupling coordination between agricultural carbon emission efficiency and food security in China: The spatial-temporal evolution and prediction
Source: PLoS One. 2025 Jul 2;20(7):e0325026. doi: 10.1371/journal.pone.0325026 (PMC12221183; doi:10.1371/journal.pone.0325026)
Supplement: S1 File — (DOCX) [file pone.0325026.s001.docx]

# Supplementary Material

## S1. Entropy-weighted TOPSIS method

Step 1: The initial step involves normalizing the raw data using the maximum and minimum method. The positive index is processed by Equation (S1), and the negative index is calculated by Equation (S2):

$X_{ij}=\frac{x_{ij}-min\left( x_{j} \right)}{\max\left( x_{j} \right)-min\left( x_{j} \right)}$ (S1)

$X_{ij}=\frac{\max\left( x_{j} \right)-x_{ij}}{\max\left( x_{j} \right)-min\left( x_{j} \right)}$ (S2)

where *X_ij_* represents the standardized value of the *j^th^* evaluation indicator for the *i^th^* evaluation object; max(*xj*) and min(*x_j_*) are the maximum and minimum values of the *j^th^* evaluation indicator, respectively.

Step 2: The second step involves calculating the proportion of the sample value of the *i^th^* evaluation object under the *j^th^* indicator.

$P_{ij}=\frac{x_{ij}^{'}}{\sum_{i=1}^{n} x_{ij}^{'}}(i=1,2,\ldots,n;j=1,2,\ldots,m)$ (S3)

Step 3: The third step is to compute the information entropy *E_j_*_​_ for the *j^th^* indicator.

$E_{j}=-k\sum_{i=1}^{n} P_{ij}\text{ h}\left( P_{ij} \right); (i=1,2,\ldots,n;j=1,2,\ldots,m)$ (S4)

Step 4: The fourth step involves calculating the diversity coefficient *D_j_* from the information entropy of the *j^th^* indicator.

$D_{j}=1-E_{j}; (j=1,2,\ldots,m)$ (S5)

Step 5: The fifth step is to calculate the weight *W_j_* for each indicator using its diversity coefficient.

$W_{j}=\frac{D_{j}}{\sum_{j=1}^{m} D_{j}}; (j=1,2,\ldots,m)$ (S6)

## S2. Individual prediction models

### GM (1,1) model

The GM model, a prominent grey prediction method, is praised for its minimal data requirements, straightforward operation, and notable accuracy. The specific steps are as follow (Qian and Wang, 2020).

Step 1: Class ratio checkout

Initially, conducting a ratio test is crucial to determine the model's applicability. Given an original time series *X*^(0)^ *= (x*^(0)^*(1), x*^(0)^*(2), …, x*^(0)^*(n))*, its class ratio *λ(t)* is computed using Equation (S8). The series is suitable for GM prediction if *λ(t)* lies within the interval *(e*^-2/(n+1)^, *e*^2/(n+2)^). Subsequently, the series undergoes a first-order accumulation generating operation (1-AGO), transforming it into the series *X*^(1)^*.*

$\lambda\left( k \right)=\frac{x^{\left( 0 \right)}\left( k-1 \right)}{x^{\left( 0 \right)}\left( k \right)}$, $k=2,\cdots,n$ (S7)

$X^{\left( 1 \right)}=\left( x^{\left( 1 \right)}\left( 1 \right),x^{\left( 1 \right)}\left( 2 \right),\cdots,x^{\left( 1 \right)}\left( k \right),\cdots,x^{\left( 1 \right)}\left( n \right) \right), x^{\left( 1 \right)}\left( k \right)=\sum_{i=1}^{k} x_{i}^{\left( 0 \right)}$ (S8)

Step 2: Model construction

The next step involves generating the nearest neighbor mean series *Z*^(1)^ = (*z*^(1)^(2), *z*^(1)^(3), ,…, *z*^(1)^(n)) from *X*^(1)^, as outlined in Equation (S10). Equations (S11) and (S12) define the first-order linear differential equation and the whitening equation. The development grey parameter *α* and the endogenous control grey parameter *μ* are ascertained using the least squares method.

$z_{k}=\frac{1}{2}\left( x^{\left( 1 \right)}\left( k \right)+x^{\left( 1 \right)}\left( k-1 \right) \right), k=2,3,\cdots,n$ (S9)

$X^{\left( 0 \right)}\left( k \right)+\alpha Z^{\left( 1 \right)}\left( k \right)=\mu,k=2,3,\cdots,n$ (S10)

$\frac{dX^{\left( 1 \right)}}{dk}+\alpha X^{\left( 1 \right)}=\mu$ (S11)

$\left( \begin{matrix} \alpha\\ \mu\end{matrix} \right)=\left( B^{T}B \right)^{-1}B^{T}Y$ (S12) $Y=\left( x^{\left( 0 \right)}\left( 2 \right),x^{\left( 0 \right)}\left( 3 \right),\cdots,x^{\left( 0 \right)}\left( n \right) \right)^{T}$ (S13)

$B=\left(\begin{matrix} \begin{matrix} -z_{2} & -z_{3} & \cdots& -z_{n} \end{matrix} \\ 1 1 \cdots{} 1 \end{matrix}\right)^{T}$ (S14)

These parameters are then integrated into the differential equation to formulate the predictive model:

$\hat{X}^{\left( 1 \right)}\left( k \right)=\left[ X^{\left( 0 \right)}\left( 1 \right)-\frac{\mu}{\alpha} \right]e^{-\alpha\left( k-1 \right)}+\frac{\mu}{\alpha}$ (S15)

Certainly, the predicted values can be derived by applying the inverse accumulated generating operation. This process can be mathematically expressed as follows:

$\hat{X}^{\left( 0 \right)}\left( k \right)=\hat{X}^{\left( 1 \right)}\left( k \right)-\hat{X}^{\left( 1 \right)}\left( k-1 \right)$ (S16)

Step 3: Precision inspection

The final stage is a residual error test to evaluate the model's predictive accuracy. The residual error *p*^(0)^(*k*) and the relative error *r*(*k*) are assessed using Equations (S18) and (S19). The model's effectiveness is determined based on criteria outlined in Table S4, ensuring a robust evaluation of the GM model's predictive capabilities.

Residual error test is applied to verifying the prediction model accuracy. Equations (S18) and (S19) can measure the residual *p*^(0)^(*k*) and relative *r*(*k*) errors, respectively.

$p^{\left( 0 \right)}\left( k \right)=\left| X^{\left( 0 \right)}\left( k \right)-\hat{X}^{\left( 0 \right)}\left( k \right) \right|, k=1,2,\cdots,n$ (S17)

$r\left( k \right)=\frac{p^{\left( 0 \right)}\left( k \right)}{X^{\left( 0 \right)}\left( k \right)}\cdot100\%, k=1,2,\cdots,n$ (S18)

### ARIMA model

ARIMA model is a classic method for time series analysis and forecasting(Alzahrani et al., 2020). Among the various model structures, the Autoregressive (AR) model stands out as one of the simplest and widely utilized ones. In the AR model, the current output “z_t_” is determined by its previous values and the parameters “α_t-p_” as depicted in Equation (S20), where “t” represents the time and "p" signifies the order of parameters.

$z_{t}=-\alpha_{1}z_{t-1}-\alpha_{2}z_{t-2}--\alpha_{k}z_{t-p}+\varepsilon$ (S19)

The expression for “α(z^−1^)” can be described as follows:

${\alpha(z}^{-1})=1-\alpha_{1}z^{-1}++ \alpha_{k}z^{-p}$ (S20)

Moving on to a less conventional model structure compared to AR, we encounter the Moving Average (MA) model. In the MA model, the output “z(t)” is defined in terms of the innovation input, which is filtered with the weights “βq,” as presented in Equations (S22) and (S23).

$z_{t}=\varepsilon+\beta_{1}\varepsilon_{t-1}+ \beta_{2}\varepsilon_{t-2} + {+\beta}_{t}\varepsilon_{t-q}$  (S21)

${\beta(z}^{-1})=1-\beta_{1}z^{-1}+ {+\beta}_{k}z^{-p}$ (S22)

When we combine both AR and MA components, we arrive at a more comprehensive model known as the Autoregressive Integrated Moving Average (ARIMA) model, as defined in Equation (S23).

$z_{t}=-\alpha_{1}z_{t-1}-{-\alpha}_{k}z_{t-p}+\varepsilon+\beta_{1}\varepsilon_{t-1}+ {+\beta}_{t}\varepsilon_{t-q}$ (S23)

Taking it a step further, the Autoregressive Integrated Moving Average (ARIMA) model incorporates differencing at least once. The formula for the ARIMA model is articulated in Equation (S24). It's worth noting that this model has demonstrated successful predictive capabilities in various practical examples across different fields, as mentioned in reference.

$z_{t}=(1-z_{t-d}){-\alpha}_{1}z_{t-1}-{-\alpha}_{p}z_{t-p}+\varepsilon+\beta_{1}\varepsilon_{t-1}+ {+\beta}_{1}\varepsilon_{t-q}$ (S24)

The ARIMA model is parameterized by specifying three model terms: “p” for AR, “q” for MA, and the number of difference steps denoted as “d.”

### LSTM model

LSTM, short for Long Short-Term Memory, is a specialized neural network architecture that is an evolution of the recurrent neural network (RNN) structure, first introduced in 1997 by Hochreiter and Schmidhuber (Hochreiter and Schmidhuber, 1997). LSTM incorporates the concept of gates, including input, output, and forgetting gates, to efficiently manage the flow of information within the neuron units, building upon the foundation of RNN. These gates, found within each LSTM unit, are activated by the sigmoid function, determining whether to allow or suppress the flow of information. The ultimate output is a result of the combined influence of the output gate and the state of the neuron unit. The relevant formulas are as follows (Liu et al., 2022):

$i_{t}=\sigma\left( W_{xi}x_{t}+W_{hi}h_{t-1}+W_{ci}c_{t-1}+b_{i} \right)$ (S25)

$f_{t}=\sigma\left( W_{xf}x_{t}+W_{hf}h_{t-1}+W_{cf}c_{t-1}+b_{f} \right)$ (S26)

$c_{t}=f_{t}\cdot c_{t-1}+i_{t}\cdot tanh \left( W_{xc}x_{t}+W_{hc}h_{t-1}+b_{c} \right)$ (S27)

$o_{t}=\sigma\left( W_{xo}x_{t}+W_{ho}h_{t-1}+W_{co}c_{t}+b_{o} \right)$ (S28)

$h_{t}=o_{t}\cdot tanh \left( c_{t} \right)$ (S38)

where *i_t_*, *f_t_*, and *o_t_* correspond to the input, forgetting, and output gates, respectively, with *σ* representing the sigmoid function. Furthermore, *c*, *W*, and *b* refer to the cell state, weight coefficients, and the bias vector, respectively, while *x_t_* and *h_t_* denote the input and output at time *t,* respectively.

In our study, the LSTM architecture comprises an input layer, one hidden layers with 100 neuron units, and an output layer. A dropout value of 0.2 is applied to mitigate overfitting. The model employs a learning rate of 0.001 and utilizes mean square error (MSE) as the chosen loss function. The training dataset spans CCD levels from 2007 to 2021, with data grouped in five-year intervals for prediction of the sixth-year data. The model parameters are continuously optimized using the Adam optimizer. Ultimately, the trained model enables the prediction of CCD levels for the years 2022to 2030.

## References

Alzahrani, S.I., Aljamaan, I.A., Al-Fakih, E.A., 2020. Forecasting the spread of the COVID-19 pandemic in Saudi Arabia using ARIMA prediction model under current public health interventions. Journal of Infection and Public Health 13(7), 914-919.

Hochreiter, S., Schmidhuber, J.J.N.c., 1997. Long short-term memory. Neural computation 9(8), 1735-1780.

Liu, J., Yu, Z., Zuo, H., Fu, R., Feng, X., 2022. Multi-stage residual life prediction of aero-engine based on real-time clustering and combined prediction model. Reliability Engineering & System Safety 225, 108624.

Qian, W., Wang, J., 2020. An improved seasonal GM(1,1) model based on the HP filter for forecasting wind power generation in China. Energy 209, 118499.

## List of Figure Captions

Fig. S1. Spatial and temporal distribution of ACEE in Chinese provinces in selected years

Fig. S2. Spatial and temporal distribution of FS in Chinese provinces in selected years

Fig. S3. The trend of U1/U2 from 2007 and 2021.

Fig. S4. CCD in northeast provinces from 2007 to 2021

Fig. S5. CCD in center provinces from 2007 to 2021

Fig. S6. CCD in western provinces from 2007 to 2021

Fig. S7. CCD in eastern provinces from 2007 to 2021

Fig. S8. The prediction of CCD in China and four major regions in selected years

## List of Table Captions

Table S1 Evaluation indexes of prediction models

Table S2 The errors of prediction models

Table S3 The weights of three prediction models from 2007 to 2021

Table S4 The weights of three prediction models from 2022 to 2030

**
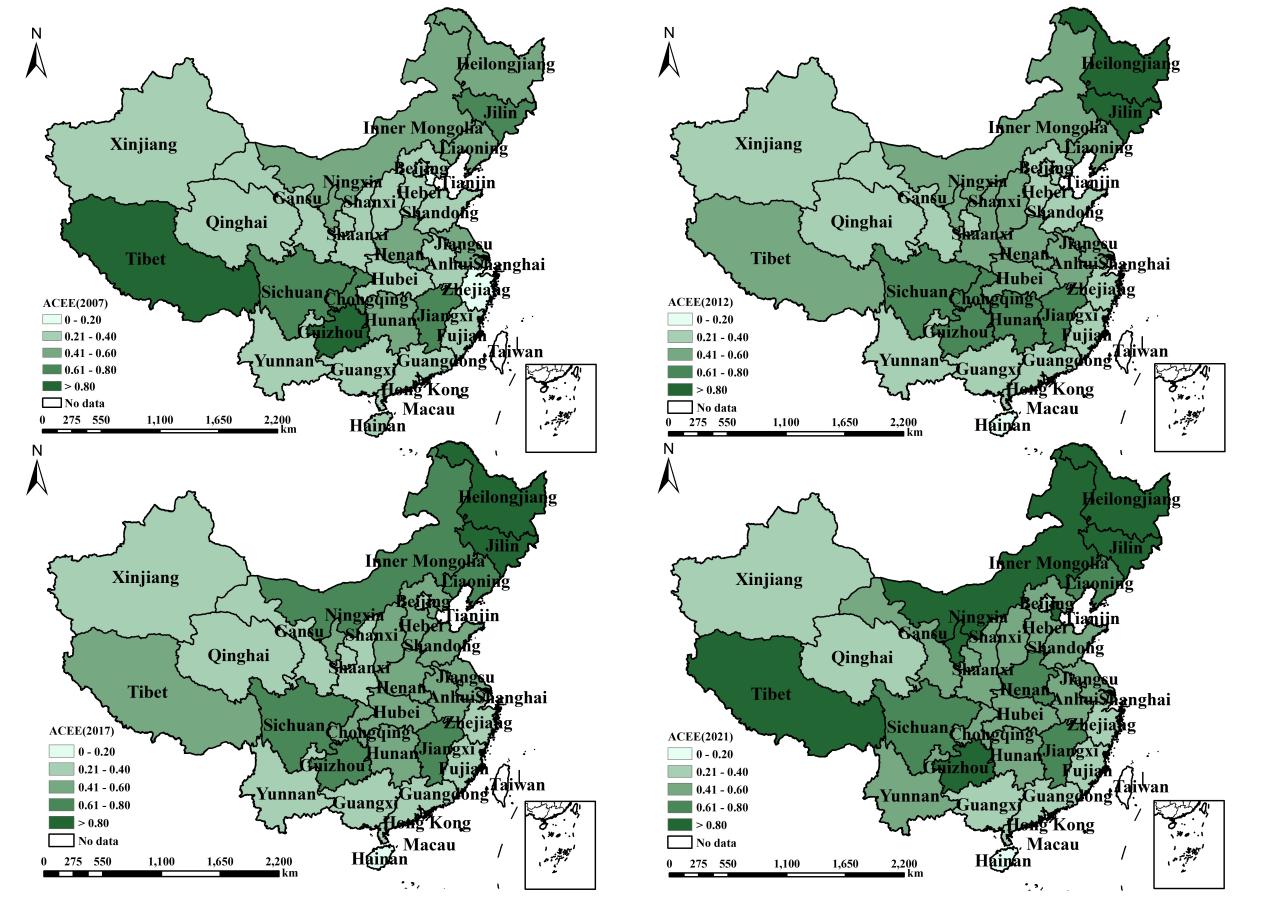
**

Fig. S1. Spatial and temporal distribution of ACEE in Chinese provinces in selected years

**
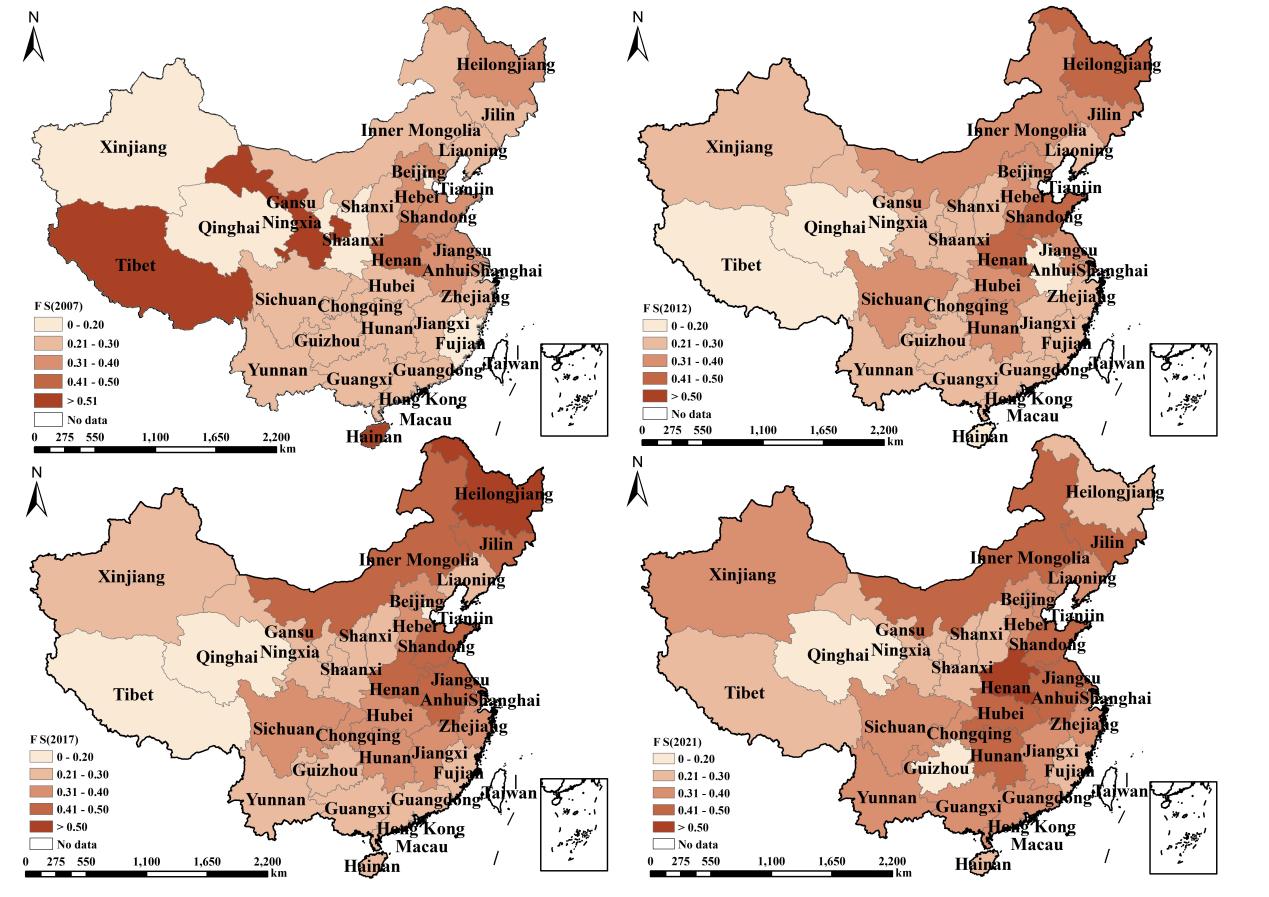
**

Fig. S2. Spatial and temporal distribution of FS in Chinese provinces in selected years


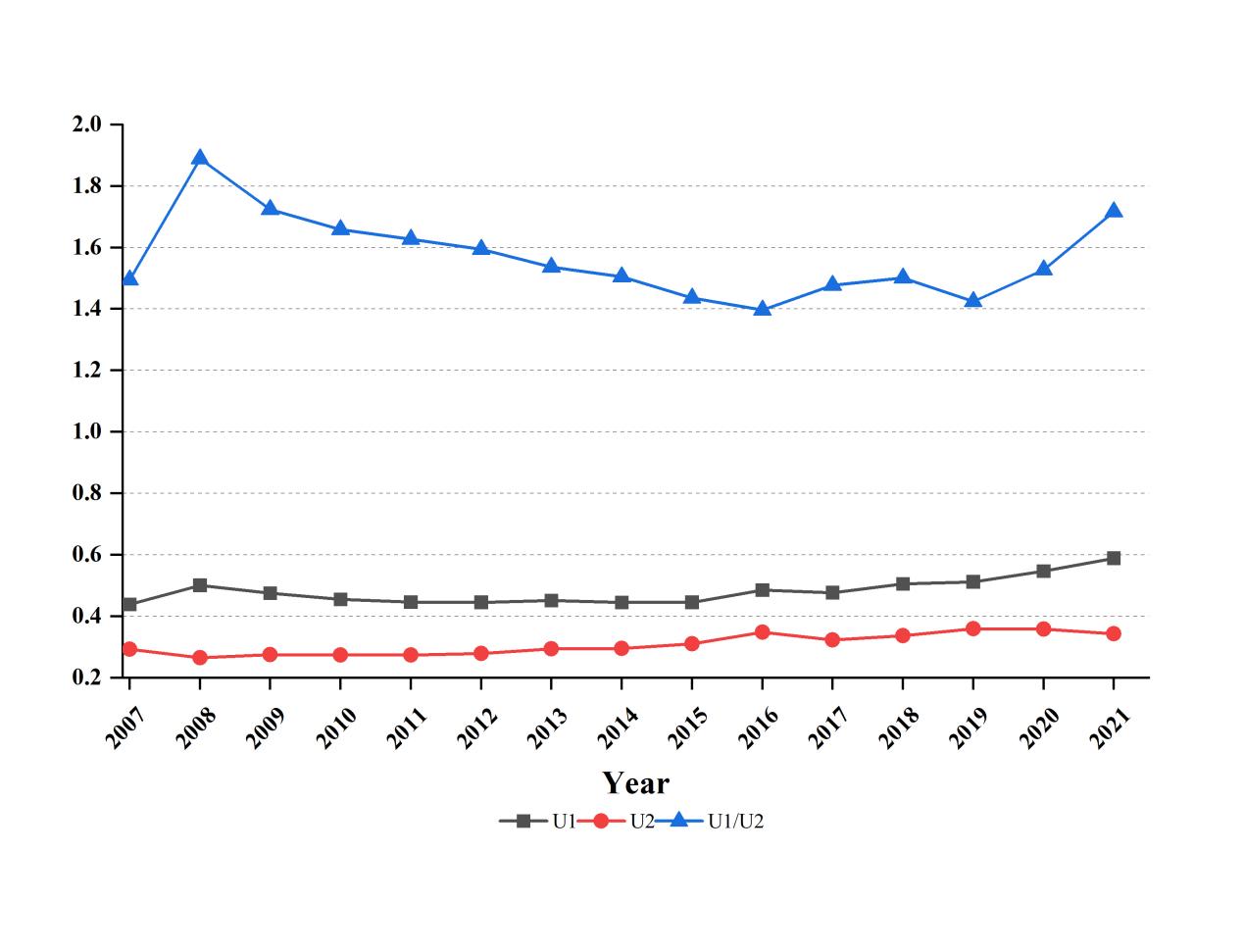


Fig. S3. The trend of U_1_/U_2_ from 2007 and 2021


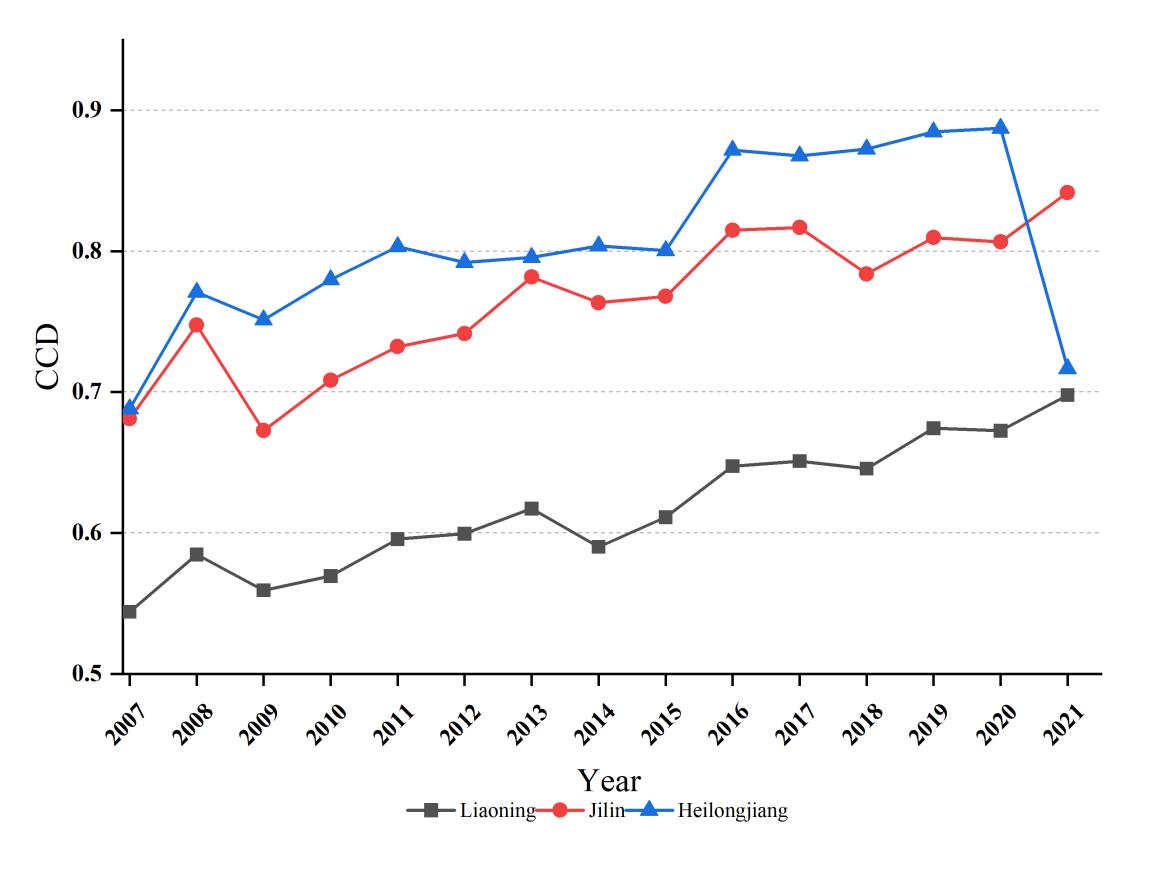


Fig. S4. CCD in northeast provinces from 2007 to 2021


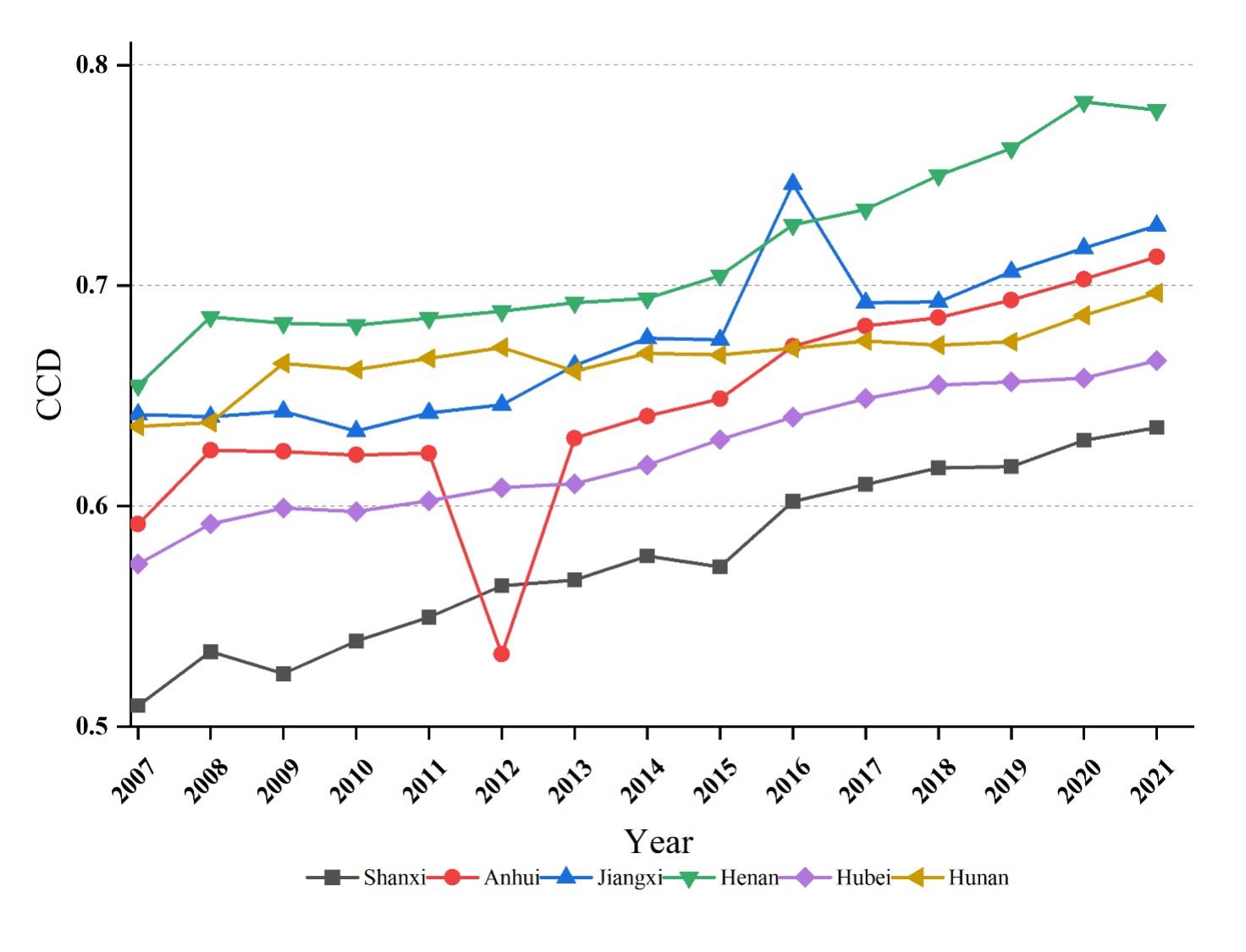


Fig. S5. CCD in center provinces from 2007 to 2021


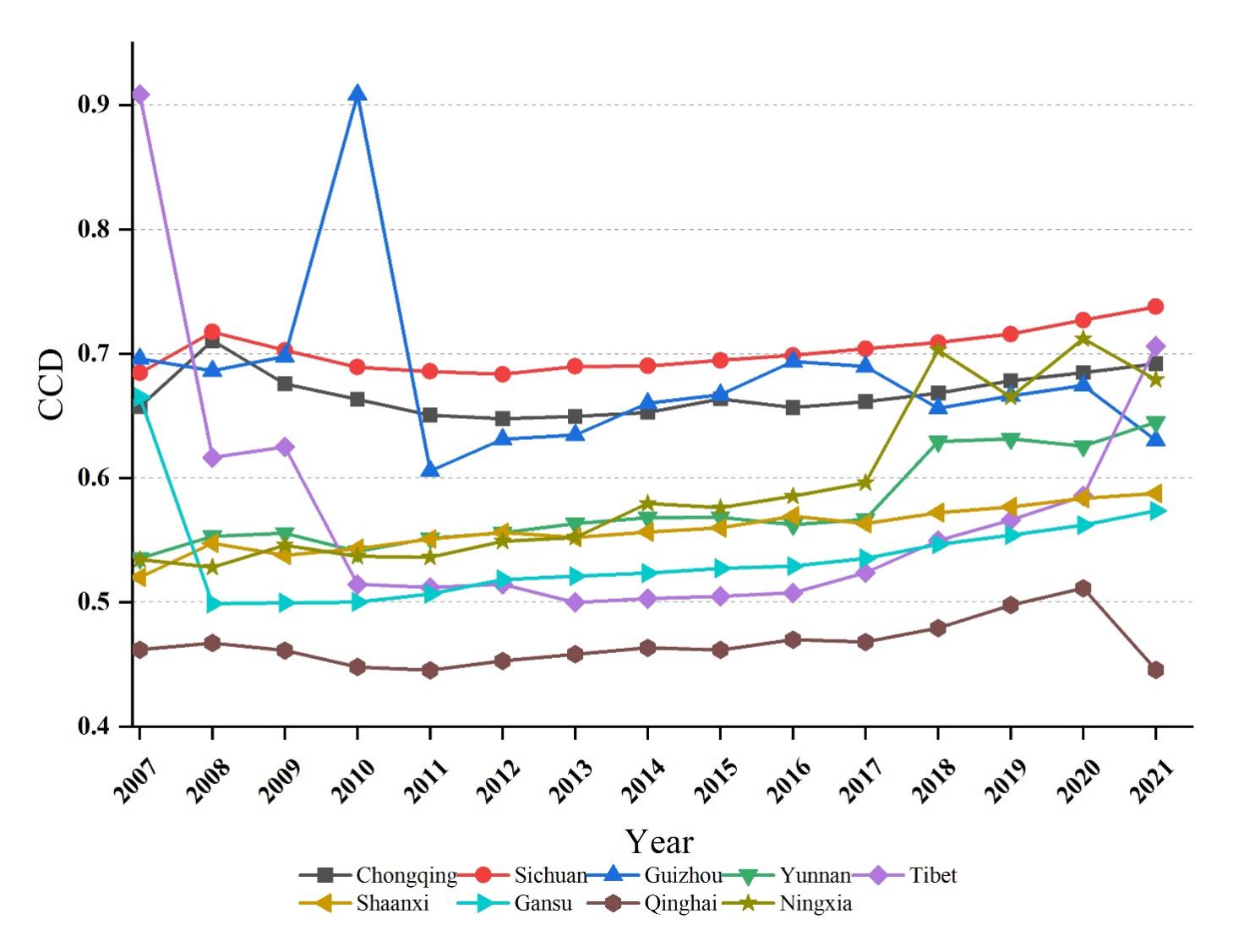


Fig. S6. CCD in western provinces from 2007 to 2021


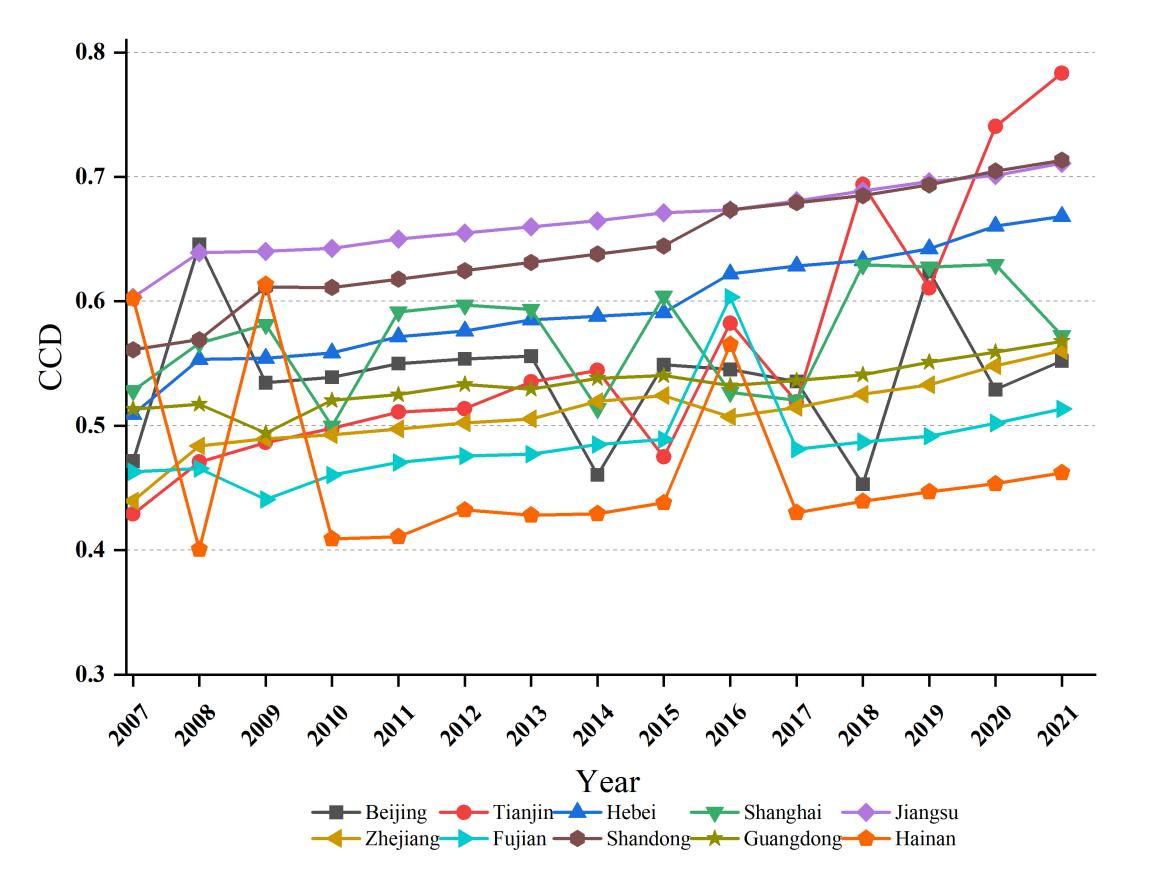


Fig. S7. CCD in eastern provinces from 2007 to 2021

**
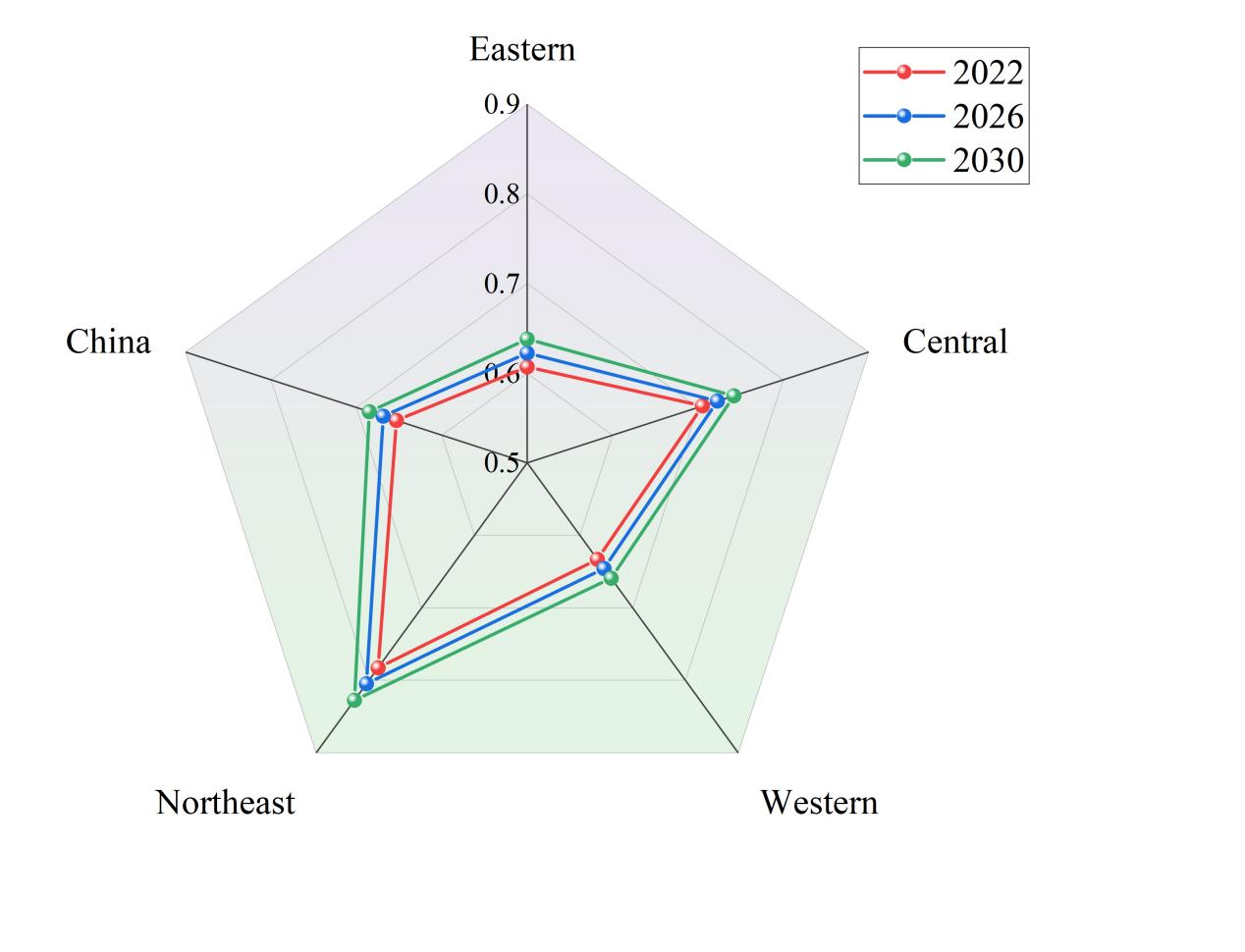
**

Fig. S8. The prediction of CCD in China and four major regions in selected years

Table S1 Evaluation indexes of prediction models

| Province | LSTM | | | GM | | | ARIMA | | |
| --- | --- | --- | --- | --- | --- | --- | --- | --- | --- |
|  | MAE | RMSE | MAPE | MAE | RMSE | MAPE | MAE | RMSE | MAPE |
| Beijing | 0.027 | 0.035 | 0.051 | 0.031 | 0.047 | 0.058 | 0.036 | 0.044 | 0.069 |
| Tianjin | 0.025 | 0.033 | 0.044 | 0.041 | 0.052 | 0.071 | 0.035 | 0.048 | 0.060 |
| Hebei | 0.005 | 0.006 | 0.008 | 0.005 | 0.006 | 0.008 | 0.007 | 0.011 | 0.013 |
| Shanxi | 0.004 | 0.000 | 0.008 | 0.005 | 0.006 | 0.009 | 0.007 | 0.008 | 0.012 |
| Inner Mongolia | 0.011 | 0.014 | 0.015 | 0.013 | 0.020 | 0.019 | 0.016 | 0.023 | 0.024 |
| Liaoning | 0.008 | 0.010 | 0.014 | 0.011 | 0.013 | 0.018 | 0.011 | 0.015 | 0.019 |
| Jilin | 0.014 | 0.016 | 0.018 | 0.018 | 0.022 | 0.023 | 0.020 | 0.027 | 0.027 |
| Heilongjiang | 0.016 | 0.024 | 0.020 | 0.031 | 0.046 | 0.039 | 0.039 | 0.053 | 0.050 |
| Shanghai | 0.027 | 0.030 | 0.049 | 0.037 | 0.040 | 0.067 | 0.037 | 0.043 | 0.066 |
| Jiangsu | 0.001 | 0.002 | 0.002 | 0.002 | 0.002 | 0.003 | 0.004 | 0.009 | 0.007 |
| Zhejiang | 0.004 | 0.005 | 0.007 | 0.006 | 0.007 | 0.011 | 0.008 | 0.012 | 0.015 |
| Anhui | 0.012 | 0.020 | 0.020 | 0.016 | 0.028 | 0.027 | 0.016 | 0.031 | 0.027 |
| Fujian | 0.015 | 0.025 | 0.029 | 0.016 | 0.031 | 0.031 | 0.022 | 0.036 | 0.043 |
| Jiangxi | 0.008 | 0.013 | 0.011 | 0.010 | 0.017 | 0.015 | 0.012 | 0.019 | 0.018 |
| Shandong | 0.005 | 0.007 | 0.007 | 0.006 | 0.008 | 0.009 | 0.006 | 0.010 | 0.010 |
| Henan | 0.004 | 0.005 | 0.005 | 0.009 | 0.012 | 0.013 | 0.009 | 0.010 | 0.012 |
| Hubei | 0.004 | 0.004 | 0.006 | 0.004 | 0.004 | 0.006 | 0.003 | 0.004 | 0.005 |
| Hunan | 0.003 | 0.004 | 0.004 | 0.006 | 0.007 | 0.009 | 0.006 | 0.008 | 0.009 |
| Guangdong | 0.004 | 0.005 | 0.007 | 0.006 | 0.008 | 0.012 | 0.007 | 0.011 | 0.013 |
| Guangxi | 0.002 | 0.003 | 0.004 | 0.004 | 0.004 | 0.007 | 0.005 | 0.006 | 0.009 |
| Hainan | 0.028 | 0.041 | 0.059 | 0.040 | 0.058 | 0.081 | 0.048 | 0.064 | 0.097 |
| Chongqing | 0.003 | 0.004 | 0.004 | 0.014 | 0.018 | 0.021 | 0.012 | 0.016 | 0.018 |
| Sichuan | 0.002 | 0.002 | 0.002 | 0.011 | 0.013 | 0.015 | 0.010 | 0.012 | 0.013 |
| Guizhou | 0.029 | 0.044 | 0.040 | 0.041 | 0.066 | 0.057 | 0.039 | 0.067 | 0.054 |
| Yunnan | 0.009 | 0.012 | 0.015 | 0.014 | 0.017 | 0.024 | 0.011 | 0.018 | 0.019 |
| Tibet | 0.014 | 0.019 | 0.024 | 0.046 | 0.058 | 0.081 | 0.062 | 0.101 | 0.097 |
| Shaanxi | 0.002 | 0.003 | 0.004 | 0.003 | 0.004 | 0.006 | 0.004 | 0.007 | 0.008 |
| Gansu | 0.003 | 0.003 | 0.005 | 0.004 | 0.005 | 0.007 | 0.029 | 0.041 | 0.051 |
| Qinghai | 0.006 | 0.010 | 0.013 | 0.012 | 0.016 | 0.025 | 0.011 | 0.016 | 0.024 |
| Ningxia | 0.012 | 0.017 | 0.020 | 0.022 | 0.026 | 0.036 | 0.017 | 0.028 | 0.028 |
| Xinjiang | 0.005 | 0.007 | 0.009 | 0.007 | 0.008 | 0.013 | 0.008 | 0.011 | 0.015 |

Table S2 The errors of prediction models

| Province | Model | 2007 | 2008 | 2009 | 2010 | 2011 | 2012 | 2013 | 2014 | 2015 | 2016 | 2017 | 2018 | 2019 | 2020 | 2021 | RSS |
| --- | --- | --- | --- | --- | --- | --- | --- | --- | --- | --- | --- | --- | --- | --- | --- | --- | --- |
| Beijing | LSTM | 0.000 | 0.037 | -0.022 | -0.015 | -0.001 | 0.018 | 0.030 | -0.058 | 0.017 | 0.013 | 0.012 | -0.069 | 0.072 | -0.018 | -0.002 | 0.034 |
|  | GM | 0.000 | 0.083 | -0.025 | -0.018 | -0.004 | 0.002 | 0.007 | -0.086 | 0.006 | 0.004 | -0.002 | -0.082 | 0.091 | -0.001 | 0.025 | 0.045 |
|  | ARIMA | -0.068 | 0.076 | 0.037 | 0.021 | 0.023 | 0.028 | 0.034 | -0.059 | -0.026 | -0.010 | -0.010 | -0.092 | 0.029 | 0.007 | 0.017 | 0.044 |
|  | CPM | 0.000 | 0.037 | -0.009 | -0.006 | 0.000 | 0.002 | 0.007 | -0.058 | 0.004 | 0.002 | -0.002 | -0.069 | 0.029 | -0.001 | 0.002 | 0.027 |
| Tianjin | LSTM | 0.000 | -0.014 | 0.002 | 0.005 | 0.004 | -0.005 | 0.013 | 0.021 | -0.055 | 0.039 | -0.045 | 0.071 | -0.054 | 0.005 | 0.018 | 0.033 |
|  | GM | 0.000 | 0.033 | 0.029 | 0.022 | 0.015 | -0.002 | -0.001 | -0.011 | -0.102 | -0.016 | -0.100 | 0.053 | -0.051 | 0.055 | 0.076 | 0.050 |
|  | ARIMA | -0.024 | -0.012 | -0.030 | -0.033 | -0.032 | -0.039 | -0.026 | -0.035 | -0.090 | 0.034 | -0.086 | 0.081 | -0.099 | 0.050 | 0.093 | 0.058 |
|  | CPM | 0.000 | -0.005 | 0.001 | 0.004 | 0.003 | -0.002 | -0.001 | -0.006 | -0.055 | 0.008 | -0.045 | 0.053 | -0.051 | 0.005 | 0.018 | 0.027 |
| Hebei | LSTM | 0.000 | -0.003 | -0.006 | 0.004 | 0.002 | -0.001 | 0.001 | -0.003 | -0.007 | 0.013 | 0.007 | -0.003 | -0.008 | -0.001 | 0.009 | 0.006 |
|  | GM | 0.000 | 0.010 | 0.002 | -0.003 | 0.002 | -0.002 | -0.002 | -0.008 | -0.015 | 0.007 | 0.003 | -0.002 | -0.002 | 0.006 | 0.003 | 0.006 |
|  | ARIMA | -0.010 | 0.007 | -0.005 | -0.010 | -0.008 | -0.012 | -0.013 | -0.018 | -0.023 | -0.003 | -0.007 | -0.012 | -0.012 | -0.004 | 0.004 | 0.011 |
|  | CPM | 0.000 | 0.002 | -0.001 | -0.001 | 0.001 | -0.001 | -0.001 | -0.003 | -0.007 | 0.002 | 0.002 | -0.002 | -0.002 | 0.000 | 0.003 | 0.003 |
| Shanxi | LSTM | 0.000 | -0.001 | -0.013 | 0.006 | 0.005 | 0.007 | 0.001 | 0.002 | -0.012 | 0.009 | 0.002 | 0.000 | -0.002 | 0.003 | 0.004 | 0.006 |
|  | GM | 0.000 | 0.008 | -0.010 | -0.003 | 0.000 | 0.005 | -0.001 | 0.001 | -0.013 | 0.008 | 0.007 | 0.005 | -0.003 | -0.001 | -0.004 | 0.006 |
|  | ARIMA | -0.009 | 0.001 | -0.020 | -0.005 | -0.007 | -0.005 | -0.012 | -0.007 | -0.017 | 0.004 | -0.009 | -0.009 | -0.014 | -0.007 | -0.001 | 0.010 |
|  | CPM | 0.000 | 0.001 | -0.010 | -0.001 | 0.000 | 0.002 | 0.000 | 0.001 | -0.012 | 0.004 | 0.001 | 0.000 | -0.002 | -0.001 | -0.001 | 0.004 |
| Inner Mongolia | LSTM | 0.000 | 0.012 | -0.019 | -0.003 | 0.006 | 0.004 | 0.016 | 0.004 | -0.013 | 0.012 | -0.004 | -0.005 | -0.024 | 0.029 | -0.005 | 0.013 |
|  | GM | 0.000 | 0.035 | -0.002 | -0.004 | 0.006 | -0.003 | 0.002 | -0.016 | -0.029 | -0.003 | -0.017 | -0.007 | -0.009 | 0.051 | -0.001 | 0.019 |
|  | ARIMA | -0.015 | -0.001 | -0.039 | -0.017 | -0.009 | -0.021 | -0.012 | -0.026 | -0.023 | 0.001 | -0.024 | -0.008 | -0.016 | 0.023 | -0.010 | 0.019 |
|  | CPM | 0.000 | 0.001 | -0.002 | -0.003 | 0.002 | -0.002 | 0.001 | -0.003 | -0.013 | 0.001 | -0.004 | -0.005 | -0.009 | 0.023 | -0.001 | 0.008 |
| Liaoning | LSTM | 0.000 | 0.009 | -0.008 | -0.002 | 0.002 | 0.004 | 0.014 | -0.018 | -0.009 | 0.016 | 0.009 | -0.008 | 0.005 | -0.010 | 0.005 | 0.009 |
|  | GM | 0.000 | 0.024 | -0.011 | -0.010 | 0.008 | 0.001 | 0.010 | -0.026 | -0.015 | 0.012 | 0.005 | -0.010 | 0.008 | -0.004 | 0.011 | 0.012 |
|  | ARIMA | -0.010 | 0.002 | -0.014 | -0.025 | -0.007 | -0.007 | -0.002 | -0.037 | -0.035 | -0.001 | 0.001 | -0.018 | -0.003 | -0.009 | 0.016 | 0.017 |
|  | CPM | 0.000 | 0.005 | -0.008 | -0.002 | 0.001 | 0.001 | 0.001 | -0.018 | -0.009 | 0.001 | 0.001 | -0.008 | 0.002 | -0.004 | 0.005 | 0.007 |
| Jilin | LSTM | 0.000 | 0.027 | -0.027 | -0.008 | 0.001 | -0.006 | 0.018 | -0.009 | -0.013 | 0.025 | 0.015 | -0.017 | 0.005 | -0.010 | 0.015 | 0.016 |
|  | GM | 0.000 | 0.039 | -0.045 | -0.019 | -0.004 | -0.004 | 0.027 | -0.002 | -0.007 | 0.031 | 0.023 | -0.020 | -0.005 | -0.018 | 0.006 | 0.022 |
|  | ARIMA | -0.010 | 0.018 | -0.048 | -0.019 | -0.007 | -0.008 | 0.017 | -0.011 | -0.016 | 0.018 | 0.008 | -0.033 | -0.017 | -0.029 | 0.006 | 0.021 |
|  | CPM | 0.000 | 0.025 | -0.027 | -0.008 | -0.001 | -0.004 | 0.017 | -0.002 | -0.007 | 0.018 | 0.008 | -0.017 | -0.002 | -0.010 | 0.006 | 0.013 |
| Heilongjiang | LSTM | 0.000 | -0.004 | -0.005 | 0.006 | 0.009 | 0.000 | -0.003 | -0.007 | -0.028 | 0.023 | -0.005 | -0.007 | 0.016 | 0.054 | -0.058 | 0.023 |
|  | GM | 0.000 | -0.003 | -0.029 | -0.007 | 0.010 | -0.007 | -0.009 | -0.007 | -0.017 | 0.049 | 0.039 | 0.036 | 0.043 | 0.039 | -0.137 | 0.044 |
|  | ARIMA | -0.100 | 0.044 | -0.026 | 0.014 | 0.020 | -0.005 | 0.005 | 0.011 | 0.003 | 0.076 | 0.029 | 0.036 | 0.045 | 0.040 | -0.132 | 0.053 |
|  | CPM | 0.000 | -0.002 | -0.005 | 0.003 | 0.009 | 0.000 | -0.002 | -0.003 | -0.002 | 0.023 | 0.004 | 0.005 | 0.016 | 0.039 | -0.058 | 0.020 |
| Shanghai | LSTM | 0.000 | 0.008 | 0.024 | -0.045 | 0.006 | 0.019 | 0.026 | -0.043 | 0.043 | -0.031 | -0.050 | 0.031 | 0.017 | 0.018 | -0.021 | 0.029 |
|  | GM | 0.000 | 0.013 | 0.024 | -0.060 | 0.029 | 0.030 | 0.024 | -0.059 | 0.027 | -0.053 | -0.063 | 0.042 | 0.037 | 0.036 | -0.024 | 0.039 |
|  | ARIMA | -0.044 | -0.006 | 0.009 | -0.073 | 0.019 | 0.025 | 0.021 | -0.058 | 0.032 | -0.045 | -0.052 | 0.057 | 0.055 | 0.057 | 0.000 | 0.043 |
|  | CPM | 0.000 | 0.003 | 0.009 | -0.045 | 0.006 | 0.019 | 0.021 | -0.043 | 0.027 | -0.031 | -0.050 | 0.031 | 0.017 | 0.018 | 0.000 | 0.026 |
| Jiangsu | LSTM | 0.000 | 0.005 | -0.001 | -0.001 | 0.002 | 0.001 | 0.000 | 0.000 | 0.003 | 0.001 | -0.002 | 0.000 | 0.002 | 0.000 | 0.004 | 0.002 |
|  | GM | 0.000 | 0.005 | 0.001 | -0.001 | 0.000 | 0.000 | -0.001 | -0.001 | -0.001 | -0.005 | -0.002 | 0.000 | 0.001 | 0.000 | 0.005 | 0.002 |
|  | ARIMA | -0.006 | 0.008 | 0.000 | -0.004 | -0.004 | -0.005 | -0.007 | -0.008 | -0.007 | -0.010 | -0.009 | -0.007 | -0.006 | -0.007 | 0.003 | 0.007 |
|  | CPM | 0.000 | 0.005 | 0.000 | -0.001 | 0.000 | 0.000 | 0.000 | 0.000 | -0.001 | -0.001 | -0.002 | 0.000 | 0.001 | 0.000 | 0.004 | 0.002 |
| Zhejiang | LSTM | 0.000 | -0.003 | 0.001 | -0.001 | 0.002 | -0.001 | -0.002 | 0.007 | 0.011 | -0.009 | -0.003 | 0.002 | -0.002 | -0.001 | 0.004 | 0.005 |
|  | GM | 0.000 | 0.001 | 0.003 | 0.001 | 0.000 | 0.000 | -0.001 | 0.007 | 0.007 | -0.015 | -0.012 | -0.007 | -0.004 | 0.006 | 0.012 | 0.007 |
|  | ARIMA | -0.009 | -0.009 | -0.009 | -0.009 | -0.009 | -0.009 | -0.009 | -0.009 | -0.009 | -0.009 | -0.009 | -0.009 | -0.009 | -0.009 | 0.003 | 0.008 |
|  | CPM | 0.000 | -0.001 | 0.001 | 0.000 | 0.000 | 0.000 | -0.001 | 0.003 | 0.003 | -0.009 | -0.003 | -0.001 | -0.002 | -0.001 | -0.002 | 0.003 |
| Anhui | LSTM | 0.000 | -0.002 | 0.007 | 0.013 | 0.019 | -0.066 | 0.018 | 0.011 | 0.003 | 0.016 | 0.002 | -0.004 | -0.005 | -0.001 | 0.003 | 0.019 |
|  | GM | 0.000 | 0.031 | 0.023 | 0.012 | 0.005 | -0.095 | -0.006 | -0.004 | -0.005 | 0.009 | 0.011 | 0.005 | 0.004 | 0.005 | 0.006 | 0.027 |
|  | ARIMA | -0.008 | 0.004 | -0.006 | -0.014 | -0.019 | -0.100 | -0.010 | -0.009 | -0.009 | 0.004 | 0.004 | -0.001 | -0.002 | -0.002 | 0.008 | 0.027 |
|  | CPM | 0.000 | 0.001 | 0.003 | 0.004 | 0.003 | -0.066 | -0.003 | -0.002 | -0.002 | 0.004 | 0.002 | -0.001 | -0.001 | -0.001 | 0.003 | 0.017 |
| Fujian | LSTM | 0.000 | 0.012 | -0.012 | 0.003 | 0.002 | 0.000 | -0.011 | -0.016 | -0.021 | 0.082 | -0.021 | -0.014 | -0.010 | 0.001 | 0.005 | 0.024 |
|  | GM | 0.000 | 0.006 | -0.022 | -0.008 | -0.003 | -0.001 | -0.005 | -0.001 | -0.002 | 0.107 | -0.019 | -0.018 | -0.018 | -0.012 | -0.006 | 0.030 |
|  | ARIMA | -0.024 | -0.021 | -0.046 | -0.027 | -0.016 | -0.011 | -0.010 | -0.002 | 0.002 | 0.116 | -0.006 | 0.000 | 0.004 | 0.015 | 0.026 | 0.035 |
|  | CPM | 0.000 | 0.004 | -0.012 | -0.002 | -0.001 | 0.000 | -0.005 | -0.001 | -0.001 | 0.082 | -0.006 | 0.000 | -0.003 | 0.000 | 0.002 | 0.022 |
| Jiangxi | LSTM | 0.000 | -0.004 | 0.004 | 0.001 | 0.002 | -0.005 | -0.004 | -0.005 | -0.017 | 0.042 | -0.005 | -0.010 | -0.003 | -0.003 | 0.002 | 0.012 |
|  | GM | 0.000 | 0.009 | 0.005 | -0.011 | -0.010 | -0.014 | -0.003 | 0.002 | -0.007 | 0.057 | -0.005 | -0.012 | -0.007 | -0.004 | -0.002 | 0.016 |
|  | ARIMA | -0.007 | -0.011 | -0.013 | -0.023 | -0.019 | -0.020 | -0.009 | -0.004 | -0.011 | 0.046 | -0.014 | -0.019 | -0.012 | -0.008 | 0.002 | 0.018 |
|  | CPM | 0.000 | -0.002 | 0.002 | -0.001 | -0.002 | -0.005 | -0.003 | -0.001 | -0.007 | 0.042 | -0.005 | -0.010 | -0.003 | -0.003 | 0.001 | 0.011 |
| Shandong | LSTM | 0.000 | -0.015 | 0.009 | 0.004 | -0.004 | 0.000 | -0.001 | -0.001 | -0.001 | 0.012 | 0.006 | 0.000 | -0.006 | -0.004 | 0.006 | 0.006 |
|  | GM | 0.000 | -0.018 | 0.015 | 0.006 | 0.003 | 0.001 | -0.003 | -0.006 | -0.009 | 0.009 | 0.005 | 0.001 | -0.001 | 0.000 | -0.003 | 0.008 |
|  | ARIMA | -0.010 | -0.011 | 0.010 | -0.003 | -0.007 | -0.011 | -0.014 | -0.016 | -0.019 | -0.001 | -0.006 | -0.011 | -0.012 | -0.011 | -0.003 | 0.011 |
|  | CPM | 0.000 | -0.011 | 0.009 | 0.001 | -0.001 | 0.000 | -0.001 | -0.001 | -0.001 | 0.001 | 0.002 | 0.000 | -0.001 | 0.000 | -0.001 | 0.004 |
| Henan | LSTM | 0.000 | -0.001 | 0.004 | 0.001 | 0.003 | 0.001 | 0.000 | -0.006 | -0.006 | 0.005 | -0.001 | 0.006 | -0.003 | 0.009 | -0.005 | 0.004 |
|  | GM | 0.000 | 0.022 | 0.011 | 0.002 | -0.003 | -0.008 | -0.012 | -0.019 | -0.016 | -0.003 | -0.004 | 0.002 | 0.005 | 0.017 | 0.005 | 0.011 |
|  | ARIMA | -0.009 | -0.009 | -0.009 | -0.009 | -0.009 | -0.009 | -0.009 | -0.009 | -0.009 | -0.009 | -0.009 | -0.009 | -0.009 | -0.009 | -0.012 | 0.009 |
|  | CPM | 0.000 | -0.001 | 0.002 | 0.001 | -0.001 | -0.001 | 0.000 | -0.006 | -0.006 | -0.001 | -0.001 | 0.001 | -0.002 | 0.004 | -0.002 | 0.003 |
| Hubei | LSTM | 0.000 | -0.006 | 0.006 | 0.005 | -0.003 | 0.000 | -0.004 | -0.002 | 0.000 | 0.003 | 0.004 | 0.005 | -0.005 | -0.005 | 0.004 | 0.004 |
|  | GM | 0.000 | 0.004 | 0.005 | -0.003 | -0.004 | -0.004 | -0.008 | -0.005 | 0.000 | 0.004 | 0.007 | 0.006 | 0.001 | -0.004 | -0.002 | 0.004 |
|  | ARIMA | -0.007 | -0.013 | -0.004 | -0.003 | -0.008 | -0.005 | -0.004 | -0.011 | -0.008 | -0.009 | -0.007 | -0.007 | -0.003 | -0.006 | 0.002 | 0.007 |
|  | CPM | 0.000 | -0.002 | 0.002 | -0.001 | -0.003 | 0.000 | -0.004 | -0.002 | 0.000 | 0.001 | 0.002 | 0.002 | -0.001 | -0.004 | 0.001 | 0.002 |
| Hunan | LSTM | 0.000 | -0.008 | 0.007 | 0.000 | 0.000 | 0.006 | -0.005 | 0.001 | 0.001 | 0.001 | 0.002 | -0.001 | -0.005 | 0.001 | 0.003 | 0.004 |
|  | GM | 0.000 | -0.015 | 0.010 | 0.004 | 0.006 | 0.009 | -0.005 | 0.000 | -0.002 | -0.002 | -0.002 | -0.006 | -0.008 | 0.001 | 0.010 | 0.007 |
|  | ARIMA | -0.004 | -0.004 | -0.004 | -0.004 | -0.004 | -0.004 | -0.004 | -0.004 | -0.004 | -0.004 | -0.004 | -0.004 | -0.004 | -0.004 | 0.006 | 0.004 |
|  | CPM | 0.000 | -0.004 | 0.002 | 0.000 | 0.000 | 0.002 | -0.004 | 0.000 | -0.001 | -0.001 | -0.001 | -0.001 | -0.004 | 0.000 | 0.003 | 0.002 |
| Guangdong | LSTM | 0.000 | 0.001 | -0.013 | 0.002 | 0.003 | 0.006 | -0.003 | 0.004 | 0.003 | -0.006 | -0.003 | -0.001 | 0.001 | 0.001 | 0.001 | 0.004 |
|  | GM | 0.000 | 0.008 | -0.019 | 0.003 | 0.004 | 0.008 | 0.001 | 0.006 | 0.004 | -0.008 | -0.008 | -0.007 | -0.001 | 0.002 | 0.007 | 0.007 |
|  | ARIMA | -0.004 | -0.004 | -0.004 | -0.004 | -0.004 | -0.004 | -0.004 | -0.004 | -0.004 | -0.004 | -0.004 | -0.004 | -0.004 | -0.004 | 0.005 | 0.004 |
|  | CPM | 0.000 | 0.001 | -0.004 | 0.001 | 0.001 | 0.002 | 0.000 | 0.001 | 0.001 | -0.006 | -0.003 | -0.001 | 0.000 | 0.000 | 0.001 | 0.002 |
| Guangxi | LSTM | 0.000 | -0.001 | 0.004 | -0.003 | -0.002 | 0.002 | 0.001 | 0.000 | 0.003 | -0.004 | 0.000 | 0.004 | -0.003 | -0.002 | 0.004 | 0.002 |
|  | GM | 0.000 | -0.001 | 0.006 | -0.004 | -0.005 | 0.003 | 0.004 | 0.001 | 0.003 | -0.006 | -0.004 | -0.001 | -0.006 | 0.000 | 0.009 | 0.004 |
|  | ARIMA | -0.003 | -0.003 | -0.003 | -0.003 | -0.003 | -0.003 | -0.003 | -0.003 | -0.003 | -0.003 | -0.003 | -0.003 | -0.003 | -0.003 | 0.008 | 0.004 |
|  | CPM | 0.000 | -0.001 | 0.001 | -0.003 | -0.002 | 0.001 | 0.001 | 0.000 | 0.001 | -0.003 | 0.000 | -0.001 | -0.003 | 0.000 | 0.004 | 0.002 |
| Hainan | LSTM | 0.000 | -0.058 | 0.097 | -0.039 | -0.005 | 0.004 | -0.009 | -0.024 | -0.019 | 0.090 | -0.020 | -0.013 | -0.009 | 0.004 | 0.003 | 0.040 |
|  | GM | 0.000 | -0.053 | 0.159 | -0.045 | -0.043 | -0.022 | -0.026 | -0.025 | -0.016 | 0.111 | -0.024 | -0.015 | -0.007 | -0.001 | 0.008 | 0.056 |
|  | ARIMA | 0.141 | 0.002 | 0.125 | 0.015 | -0.073 | -0.051 | -0.046 | -0.047 | -0.037 | 0.094 | 0.015 | -0.036 | -0.024 | -0.014 | -0.002 | 0.064 |
|  | CPM | 0.000 | -0.002 | 0.097 | -0.009 | -0.005 | -0.003 | -0.009 | -0.024 | -0.016 | 0.090 | -0.006 | -0.013 | -0.007 | -0.001 | 0.001 | 0.035 |
| Chongqing | LSTM | 0.000 | 0.013 | 0.000 | -0.001 | -0.003 | -0.002 | 0.001 | 0.001 | 0.007 | -0.003 | -0.001 | 0.000 | 0.003 | 0.001 | -0.001 | 0.004 |
|  | GM | 0.000 | 0.045 | 0.010 | -0.003 | -0.016 | -0.019 | -0.018 | -0.015 | -0.004 | -0.012 | -0.008 | -0.002 | 0.008 | 0.014 | 0.021 | 0.017 |
|  | ARIMA | -0.011 | 0.047 | -0.011 | -0.008 | -0.015 | -0.013 | -0.010 | -0.007 | 0.002 | -0.010 | -0.002 | 0.003 | 0.010 | 0.012 | 0.016 | 0.016 |
|  | CPM | 0.000 | 0.013 | 0.000 | -0.001 | -0.003 | -0.002 | -0.001 | -0.001 | 0.001 | -0.003 | -0.001 | 0.000 | 0.003 | 0.001 | 0.001 | 0.004 |
| Sichuan | LSTM | 0.000 | 0.004 | 0.005 | -0.002 | 0.000 | -0.002 | 0.003 | 0.000 | 0.001 | 0.001 | 0.001 | 0.001 | -0.001 | -0.001 | 0.002 | 0.002 |
|  | GM | 0.000 | 0.030 | 0.013 | -0.004 | -0.009 | -0.014 | -0.010 | -0.012 | -0.009 | -0.008 | -0.005 | -0.002 | 0.002 | 0.011 | 0.019 | 0.012 |
|  | ARIMA | -0.022 | 0.030 | -0.013 | -0.014 | -0.006 | -0.005 | 0.003 | -0.002 | 0.002 | 0.002 | 0.004 | 0.005 | 0.007 | 0.013 | 0.014 | 0.012 |
|  | CPM | 0.000 | 0.009 | 0.003 | -0.002 | 0.000 | -0.002 | 0.001 | 0.000 | 0.001 | 0.001 | 0.001 | 0.001 | 0.001 | 0.001 | 0.002 | 0.003 |
| Guizhou | LSTM | 0.000 | -0.023 | -0.034 | 0.135 | -0.071 | -0.033 | -0.021 | 0.000 | 0.001 | 0.025 | 0.014 | -0.009 | -0.005 | 0.017 | -0.019 | 0.043 |
|  | GM | 0.000 | -0.026 | -0.009 | 0.206 | -0.091 | -0.060 | -0.051 | -0.021 | -0.009 | 0.023 | 0.024 | -0.005 | 0.011 | 0.024 | -0.015 | 0.064 |
|  | ARIMA | 0.016 | 0.006 | 0.018 | 0.229 | -0.074 | -0.049 | -0.045 | -0.020 | -0.013 | 0.014 | 0.010 | -0.024 | -0.014 | -0.005 | -0.050 | 0.067 |
|  | CPM | 0.000 | -0.004 | -0.005 | 0.135 | -0.071 | -0.033 | -0.021 | 0.000 | -0.001 | 0.014 | 0.010 | -0.009 | -0.003 | 0.003 | -0.019 | 0.041 |
| Yunnan | LSTM | 0.000 | 0.002 | 0.007 | -0.008 | -0.001 | 0.000 | 0.006 | 0.006 | 0.000 | -0.019 | -0.016 | 0.029 | 0.015 | -0.008 | -0.004 | 0.011 |
|  | GM | 0.000 | 0.021 | 0.017 | -0.005 | -0.002 | -0.004 | -0.005 | -0.007 | -0.015 | -0.028 | -0.031 | 0.023 | 0.017 | 0.004 | 0.015 | 0.016 |
|  | ARIMA | -0.008 | -0.008 | -0.008 | -0.008 | -0.008 | -0.008 | -0.008 | -0.008 | -0.008 | -0.008 | -0.008 | -0.008 | -0.008 | -0.008 | 0.011 | 0.008 |
|  | CPM | 0.000 | 0.002 | 0.003 | -0.005 | -0.001 | 0.000 | -0.002 | -0.002 | 0.000 | -0.008 | -0.008 | 0.005 | 0.004 | -0.002 | 0.003 | 0.004 |
| Tibet | LSTM | 0.000 | -0.022 | 0.046 | -0.012 | -0.012 | -0.001 | -0.007 | 0.003 | -0.003 | -0.002 | 0.002 | 0.021 | -0.012 | -0.031 | 0.035 | 0.020 |
|  | GM | 0.000 | 0.086 | 0.092 | -0.023 | -0.028 | -0.029 | -0.047 | -0.047 | -0.049 | -0.049 | -0.037 | -0.014 | -0.001 | 0.014 | 0.132 | 0.056 |
|  | ARIMA | 0.254 | -0.240 | 0.001 | -0.117 | -0.031 | -0.027 | -0.043 | -0.029 | -0.029 | -0.028 | -0.014 | -0.001 | -0.005 | 0.001 | 0.106 | 0.101 |
|  | CPM | 0.000 | -0.016 | 0.001 | -0.012 | -0.012 | -0.001 | -0.007 | -0.002 | -0.003 | -0.002 | -0.002 | -0.001 | -0.001 | 0.001 | 0.035 | 0.011 |
| Shaanxi | LSTM | 0.000 | 0.003 | -0.003 | -0.002 | 0.002 | 0.003 | -0.002 | -0.001 | -0.002 | 0.005 | -0.002 | 0.003 | 0.002 | 0.001 | -0.001 | 0.002 |
|  | GM | 0.000 | 0.009 | -0.004 | -0.003 | 0.002 | 0.004 | -0.004 | -0.002 | -0.003 | 0.003 | -0.007 | -0.001 | 0.000 | 0.004 | 0.004 | 0.004 |
|  | ARIMA | -0.004 | 0.008 | -0.005 | -0.004 | -0.001 | 0.000 | -0.007 | -0.006 | -0.006 | -0.001 | -0.010 | -0.005 | -0.004 | -0.001 | 0.002 | 0.005 |
|  | CPM | 0.000 | 0.003 | -0.003 | -0.003 | 0.000 | 0.000 | -0.002 | -0.001 | -0.002 | 0.001 | -0.002 | -0.001 | 0.000 | 0.000 | 0.001 | 0.002 |
| Gansu | LSTM | 0.000 | 0.004 | 0.003 | -0.007 | -0.005 | 0.000 | 0.001 | 0.002 | 0.002 | -0.002 | -0.002 | 0.003 | 0.002 | -0.001 | -0.003 | 0.003 |
|  | GM | 0.000 | 0.007 | 0.002 | -0.003 | -0.002 | 0.004 | 0.001 | -0.002 | -0.004 | -0.007 | -0.007 | -0.002 | 0.001 | 0.003 | 0.009 | 0.004 |
|  | ARIMA | 0.128 | -0.039 | -0.038 | -0.037 | -0.031 | -0.019 | -0.016 | -0.014 | -0.010 | -0.008 | -0.002 | 0.009 | 0.017 | 0.025 | 0.036 | 0.041 |
|  | CPM | 0.000 | 0.002 | 0.001 | -0.007 | -0.002 | 0.000 | 0.000 | -0.001 | -0.001 | -0.002 | -0.002 | 0.001 | 0.001 | 0.001 | 0.002 | 0.002 |
| Qinghai | LSTM | 0.000 | 0.003 | 0.003 | -0.003 | -0.001 | -0.001 | 0.001 | 0.003 | -0.003 | 0.001 | -0.006 | -0.005 | 0.008 | 0.025 | -0.024 | 0.010 |
|  | GM | 0.000 | 0.016 | 0.007 | -0.008 | -0.013 | -0.008 | -0.005 | -0.002 | -0.006 | 0.000 | -0.004 | 0.004 | 0.021 | 0.032 | -0.036 | 0.015 |
|  | ARIMA | -0.003 | 0.004 | -0.005 | -0.014 | -0.012 | -0.006 | -0.004 | 0.001 | -0.003 | 0.007 | 0.000 | 0.015 | 0.025 | 0.033 | -0.037 | 0.016 |
|  | CPM | 0.000 | 0.003 | 0.002 | -0.003 | -0.001 | -0.001 | 0.000 | 0.000 | -0.003 | 0.000 | 0.000 | 0.002 | 0.008 | 0.025 | -0.024 | 0.009 |
| Ningxia | LSTM | 0.000 | 0.003 | 0.010 | -0.001 | -0.016 | -0.001 | 0.000 | 0.017 | -0.005 | -0.005 | -0.018 | 0.047 | -0.015 | 0.019 | -0.013 | 0.016 |
|  | GM | 0.000 | 0.023 | 0.028 | 0.005 | -0.010 | -0.011 | -0.022 | -0.009 | -0.026 | -0.031 | -0.035 | 0.057 | 0.005 | 0.037 | -0.011 | 0.025 |
|  | ARIMA | -0.012 | -0.022 | -0.009 | -0.024 | -0.019 | -0.011 | -0.017 | -0.003 | -0.021 | -0.014 | -0.013 | 0.041 | -0.040 | 0.008 | -0.025 | 0.021 |
|  | CPM | 0.000 | 0.003 | 0.004 | -0.001 | -0.010 | -0.001 | 0.000 | -0.002 | -0.005 | -0.005 | -0.013 | 0.041 | -0.003 | 0.008 | -0.011 | 0.012 |
| Xinjiang | LSTM | 0.000 | -0.016 | 0.014 | 0.001 | 0.000 | 0.000 | 0.004 | -0.006 | 0.004 | 0.004 | 0.000 | -0.001 | -0.002 | -0.005 | 0.008 | 0.007 |
|  | GM | 0.000 | -0.017 | 0.012 | 0.004 | 0.006 | 0.003 | 0.007 | -0.007 | 0.002 | 0.001 | -0.009 | -0.008 | -0.006 | -0.002 | 0.014 | 0.008 |
|  | ARIMA | -0.008 | -0.008 | -0.008 | -0.008 | -0.008 | -0.008 | -0.008 | -0.008 | -0.008 | -0.008 | -0.008 | -0.008 | -0.008 | -0.008 | 0.013 | 0.009 |
|  | CPM | 0.000 | -0.008 | 0.004 | 0.001 | 0.000 | 0.000 | 0.002 | -0.006 | 0.002 | 0.001 | 0.000 | -0.001 | -0.002 | -0.005 | 0.008 | 0.004 |

Note: CPM-Combination prediction model, RSS-Residual square sum.

Table S3 The weights of three prediction models from 2007 to 2021

| Province | Model | 2007 | 2008 | 2009 | 2010 | 2011 | 2012 | 2013 | 2014 | 2015 | 2016 | 2017 | 2018 | 2019 | 2020 | 2021 |
| --- | --- | --- | --- | --- | --- | --- | --- | --- | --- | --- | --- | --- | --- | --- | --- | --- |
| Beijing | LSTM | 1.000 | 0.516 | 0.407 | 0.393 | 0.863 | 0.084 | 0.161 | 0.374 | 0.224 | 0.182 | 0.142 | 0.385 | 0.233 | 0.043 | 0.813 |
|  | GM | 0.000 | 0.232 | 0.352 | 0.328 | 0.117 | 0.863 | 0.696 | 0.254 | 0.631 | 0.575 | 0.688 | 0.325 | 0.184 | 0.845 | 0.076 |
|  | ARIMA | 0.000 | 0.252 | 0.241 | 0.279 | 0.020 | 0.053 | 0.143 | 0.371 | 0.146 | 0.243 | 0.171 | 0.289 | 0.583 | 0.112 | 0.112 |
| Tianjin | LSTM | 1.000 | 0.382 | 0.907 | 0.710 | 0.722 | 0.297 | 0.057 | 0.294 | 0.463 | 0.215 | 0.504 | 0.310 | 0.383 | 0.847 | 0.695 |
|  | GM | 0.000 | 0.164 | 0.047 | 0.175 | 0.189 | 0.664 | 0.916 | 0.531 | 0.251 | 0.539 | 0.229 | 0.418 | 0.407 | 0.072 | 0.167 |
|  | ARIMA | 0.000 | 0.454 | 0.046 | 0.115 | 0.088 | 0.039 | 0.028 | 0.176 | 0.285 | 0.246 | 0.267 | 0.272 | 0.210 | 0.081 | 0.138 |
| Hebei | LSTM | 1.000 | 0.559 | 0.208 | 0.353 | 0.464 | 0.667 | 0.595 | 0.630 | 0.564 | 0.140 | 0.232 | 0.359 | 0.165 | 0.818 | 0.156 |
|  | GM | 0.000 | 0.181 | 0.538 | 0.517 | 0.403 | 0.288 | 0.352 | 0.257 | 0.264 | 0.264 | 0.521 | 0.540 | 0.726 | 0.074 | 0.433 |
|  | ARIMA | 0.000 | 0.260 | 0.255 | 0.130 | 0.133 | 0.045 | 0.053 | 0.113 | 0.172 | 0.596 | 0.247 | 0.100 | 0.109 | 0.108 | 0.411 |
| Shanxi | LSTM | 1.000 | 0.482 | 0.332 | 0.249 | 0.071 | 0.251 | 0.379 | 0.399 | 0.376 | 0.242 | 0.688 | 0.898 | 0.542 | 0.261 | 0.154 |
|  | GM | 0.000 | 0.069 | 0.447 | 0.448 | 0.881 | 0.388 | 0.596 | 0.505 | 0.360 | 0.267 | 0.179 | 0.065 | 0.373 | 0.625 | 0.134 |
|  | ARIMA | 0.000 | 0.450 | 0.221 | 0.303 | 0.048 | 0.361 | 0.025 | 0.096 | 0.264 | 0.491 | 0.132 | 0.037 | 0.085 | 0.114 | 0.712 |
| Inner Mongolia | LSTM | 1.000 | 0.048 | 0.090 | 0.548 | 0.379 | 0.386 | 0.081 | 0.694 | 0.496 | 0.070 | 0.714 | 0.436 | 0.198 | 0.355 | 0.115 |
|  | GM | 0.000 | 0.017 | 0.866 | 0.359 | 0.382 | 0.542 | 0.815 | 0.189 | 0.223 | 0.311 | 0.168 | 0.298 | 0.507 | 0.203 | 0.832 |
|  | ARIMA | 0.000 | 0.935 | 0.044 | 0.093 | 0.239 | 0.072 | 0.104 | 0.118 | 0.281 | 0.619 | 0.118 | 0.266 | 0.294 | 0.442 | 0.053 |
| Liaoning | LSTM | 0.999 | 0.184 | 0.420 | 0.750 | 0.625 | 0.255 | 0.108 | 0.454 | 0.531 | 0.039 | 0.100 | 0.460 | 0.314 | 0.230 | 0.555 |
|  | GM | 0.001 | 0.071 | 0.334 | 0.182 | 0.176 | 0.617 | 0.146 | 0.321 | 0.330 | 0.055 | 0.189 | 0.343 | 0.187 | 0.516 | 0.267 |
|  | ARIMA | 0.000 | 0.744 | 0.247 | 0.069 | 0.199 | 0.129 | 0.746 | 0.225 | 0.139 | 0.906 | 0.711 | 0.197 | 0.498 | 0.254 | 0.178 |
| Jilin | LSTM | 1.000 | 0.313 | 0.459 | 0.541 | 0.690 | 0.311 | 0.363 | 0.130 | 0.271 | 0.314 | 0.271 | 0.431 | 0.445 | 0.520 | 0.169 |
|  | GM | 0.000 | 0.218 | 0.279 | 0.235 | 0.199 | 0.443 | 0.251 | 0.760 | 0.505 | 0.250 | 0.183 | 0.355 | 0.423 | 0.294 | 0.433 |
|  | ARIMA | 0.000 | 0.468 | 0.262 | 0.224 | 0.112 | 0.246 | 0.386 | 0.111 | 0.224 | 0.436 | 0.546 | 0.214 | 0.132 | 0.187 | 0.398 |
| Heilongjiang | LSTM | 1.000 | 0.416 | 0.747 | 0.450 | 0.428 | 0.898 | 0.531 | 0.390 | 0.077 | 0.563 | 0.770 | 0.708 | 0.585 | 0.270 | 0.536 |
|  | GM | 0.000 | 0.545 | 0.121 | 0.362 | 0.380 | 0.044 | 0.166 | 0.373 | 0.133 | 0.266 | 0.098 | 0.145 | 0.213 | 0.371 | 0.227 |
|  | ARIMA | 0.000 | 0.039 | 0.132 | 0.188 | 0.192 | 0.058 | 0.303 | 0.237 | 0.790 | 0.170 | 0.132 | 0.147 | 0.201 | 0.359 | 0.237 |
| Shanghai | LSTM | 1.000 | 0.339 | 0.222 | 0.425 | 0.649 | 0.414 | 0.303 | 0.407 | 0.254 | 0.441 | 0.365 | 0.439 | 0.573 | 0.545 | 0.019 |
|  | GM | 0.000 | 0.201 | 0.215 | 0.314 | 0.142 | 0.266 | 0.332 | 0.294 | 0.404 | 0.257 | 0.285 | 0.322 | 0.255 | 0.281 | 0.016 |
|  | ARIMA | 0.000 | 0.460 | 0.563 | 0.261 | 0.209 | 0.321 | 0.365 | 0.299 | 0.342 | 0.302 | 0.350 | 0.239 | 0.172 | 0.174 | 0.965 |
| Jiangsu | LSTM | 1.000 | 0.384 | 0.060 | 0.641 | 0.013 | 0.087 | 0.943 | 0.751 | 0.231 | 0.768 | 0.504 | 0.957 | 0.367 | 0.067 | 0.331 |
|  | GM | 0.000 | 0.386 | 0.036 | 0.272 | 0.980 | 0.891 | 0.048 | 0.212 | 0.681 | 0.161 | 0.393 | 0.041 | 0.526 | 0.931 | 0.245 |
|  | ARIMA | 0.000 | 0.231 | 0.904 | 0.087 | 0.007 | 0.022 | 0.009 | 0.037 | 0.088 | 0.071 | 0.102 | 0.002 | 0.108 | 0.002 | 0.424 |
| Zhejiang | LSTM | 1.000 | 0.204 | 0.649 | 0.512 | 0.142 | 0.315 | 0.342 | 0.350 | 0.267 | 0.366 | 0.656 | 0.696 | 0.538 | 0.790 | 0.384 |
|  | GM | 0.000 | 0.736 | 0.269 | 0.445 | 0.821 | 0.665 | 0.563 | 0.348 | 0.399 | 0.232 | 0.142 | 0.170 | 0.314 | 0.121 | 0.135 |
|  | ARIMA | 0.000 | 0.060 | 0.082 | 0.043 | 0.037 | 0.020 | 0.095 | 0.302 | 0.334 | 0.402 | 0.202 | 0.134 | 0.148 | 0.088 | 0.482 |
| Anhui | LSTM | 1.000 | 0.595 | 0.401 | 0.339 | 0.173 | 0.426 | 0.179 | 0.212 | 0.497 | 0.160 | 0.597 | 0.206 | 0.252 | 0.582 | 0.501 |
|  | GM | 0.000 | 0.046 | 0.127 | 0.357 | 0.657 | 0.294 | 0.513 | 0.532 | 0.318 | 0.271 | 0.109 | 0.170 | 0.269 | 0.125 | 0.286 |
|  | ARIMA | 0.000 | 0.359 | 0.472 | 0.304 | 0.170 | 0.280 | 0.308 | 0.256 | 0.184 | 0.569 | 0.294 | 0.624 | 0.478 | 0.292 | 0.213 |
| Fujian | LSTM | 1.000 | 0.296 | 0.557 | 0.651 | 0.486 | 0.928 | 0.227 | 0.044 | 0.045 | 0.404 | 0.172 | 0.000 | 0.256 | 0.925 | 0.490 |
|  | GM | 0.000 | 0.541 | 0.299 | 0.270 | 0.446 | 0.065 | 0.516 | 0.625 | 0.452 | 0.310 | 0.193 | 0.000 | 0.152 | 0.042 | 0.421 |
|  | ARIMA | 0.000 | 0.163 | 0.144 | 0.078 | 0.068 | 0.008 | 0.257 | 0.331 | 0.503 | 0.286 | 0.635 | 1.000 | 0.592 | 0.033 | 0.090 |
| Jiangxi | LSTM | 1.000 | 0.539 | 0.461 | 0.856 | 0.760 | 0.604 | 0.352 | 0.209 | 0.192 | 0.381 | 0.417 | 0.422 | 0.596 | 0.474 | 0.363 |
|  | GM | 0.000 | 0.249 | 0.388 | 0.098 | 0.159 | 0.231 | 0.472 | 0.519 | 0.508 | 0.278 | 0.434 | 0.352 | 0.260 | 0.357 | 0.298 |
|  | ARIMA | 0.000 | 0.212 | 0.151 | 0.046 | 0.082 | 0.164 | 0.176 | 0.272 | 0.299 | 0.341 | 0.149 | 0.226 | 0.144 | 0.169 | 0.339 |
| Shandong | LSTM | 1.000 | 0.319 | 0.398 | 0.327 | 0.345 | 0.655 | 0.766 | 0.884 | 0.850 | 0.087 | 0.309 | 0.753 | 0.181 | 0.094 | 0.176 |
|  | GM | 0.000 | 0.265 | 0.241 | 0.208 | 0.469 | 0.328 | 0.195 | 0.085 | 0.104 | 0.112 | 0.374 | 0.230 | 0.731 | 0.875 | 0.409 |
|  | ARIMA | 0.000 | 0.416 | 0.360 | 0.465 | 0.185 | 0.017 | 0.040 | 0.031 | 0.047 | 0.800 | 0.317 | 0.018 | 0.088 | 0.031 | 0.415 |
| Henan | LSTM | 1.000 | 0.908 | 0.543 | 0.531 | 0.444 | 0.786 | 0.946 | 0.506 | 0.491 | 0.266 | 0.716 | 0.225 | 0.539 | 0.396 | 0.387 |
|  | GM | 0.000 | 0.027 | 0.205 | 0.380 | 0.421 | 0.116 | 0.023 | 0.158 | 0.179 | 0.570 | 0.189 | 0.631 | 0.290 | 0.206 | 0.447 |
|  | ARIMA | 0.000 | 0.065 | 0.251 | 0.089 | 0.134 | 0.099 | 0.031 | 0.336 | 0.330 | 0.163 | 0.095 | 0.144 | 0.171 | 0.398 | 0.166 |
| Hubei | LSTM | 1.000 | 0.338 | 0.270 | 0.208 | 0.494 | 0.973 | 0.433 | 0.673 | 0.300 | 0.527 | 0.459 | 0.384 | 0.155 | 0.332 | 0.172 |
|  | GM | 0.000 | 0.507 | 0.307 | 0.442 | 0.354 | 0.016 | 0.202 | 0.216 | 0.690 | 0.320 | 0.268 | 0.326 | 0.571 | 0.411 | 0.366 |
|  | ARIMA | 0.000 | 0.155 | 0.423 | 0.350 | 0.152 | 0.011 | 0.365 | 0.111 | 0.010 | 0.153 | 0.272 | 0.291 | 0.274 | 0.257 | 0.462 |
| Hunan | LSTM | 1.000 | 0.298 | 0.311 | 0.952 | 0.856 | 0.343 | 0.317 | 0.170 | 0.674 | 0.538 | 0.416 | 0.691 | 0.373 | 0.547 | 0.513 |
|  | GM | 0.000 | 0.157 | 0.211 | 0.025 | 0.061 | 0.215 | 0.325 | 0.776 | 0.208 | 0.297 | 0.392 | 0.130 | 0.228 | 0.339 | 0.184 |
|  | ARIMA | 0.000 | 0.546 | 0.478 | 0.023 | 0.084 | 0.443 | 0.358 | 0.053 | 0.118 | 0.165 | 0.192 | 0.180 | 0.399 | 0.115 | 0.303 |
| Guangdong | LSTM | 1.000 | 0.731 | 0.203 | 0.421 | 0.371 | 0.306 | 0.119 | 0.384 | 0.396 | 0.304 | 0.435 | 0.700 | 0.557 | 0.707 | 0.695 |
|  | GM | 0.000 | 0.086 | 0.136 | 0.312 | 0.312 | 0.225 | 0.779 | 0.239 | 0.290 | 0.232 | 0.189 | 0.107 | 0.365 | 0.186 | 0.124 |
|  | ARIMA | 0.000 | 0.183 | 0.661 | 0.267 | 0.318 | 0.469 | 0.102 | 0.377 | 0.313 | 0.464 | 0.376 | 0.193 | 0.078 | 0.107 | 0.181 |
| Guangxi | LSTM | 1.000 | 0.394 | 0.337 | 0.385 | 0.430 | 0.470 | 0.559 | 0.804 | 0.366 | 0.355 | 0.923 | 0.220 | 0.417 | 0.055 | 0.531 |
|  | GM | 0.000 | 0.492 | 0.230 | 0.266 | 0.230 | 0.290 | 0.184 | 0.136 | 0.327 | 0.224 | 0.031 | 0.523 | 0.203 | 0.911 | 0.222 |
|  | ARIMA | 0.000 | 0.115 | 0.434 | 0.349 | 0.340 | 0.240 | 0.257 | 0.060 | 0.307 | 0.420 | 0.045 | 0.256 | 0.380 | 0.034 | 0.247 |
| Hainan | LSTM | 1.000 | 0.033 | 0.421 | 0.226 | 0.836 | 0.800 | 0.651 | 0.401 | 0.373 | 0.361 | 0.311 | 0.448 | 0.391 | 0.143 | 0.409 |
|  | GM | 0.000 | 0.036 | 0.255 | 0.198 | 0.104 | 0.140 | 0.223 | 0.391 | 0.439 | 0.292 | 0.266 | 0.391 | 0.469 | 0.820 | 0.133 |
|  | ARIMA | 0.000 | 0.931 | 0.324 | 0.576 | 0.061 | 0.059 | 0.126 | 0.208 | 0.188 | 0.346 | 0.423 | 0.161 | 0.139 | 0.037 | 0.458 |
| Chongqing | LSTM | 1.000 | 0.636 | 0.949 | 0.671 | 0.719 | 0.809 | 0.911 | 0.850 | 0.182 | 0.638 | 0.491 | 0.809 | 0.589 | 0.892 | 0.929 |
|  | GM | 0.000 | 0.185 | 0.027 | 0.249 | 0.136 | 0.076 | 0.031 | 0.048 | 0.282 | 0.158 | 0.091 | 0.123 | 0.225 | 0.051 | 0.031 |
|  | ARIMA | 0.000 | 0.179 | 0.024 | 0.081 | 0.145 | 0.115 | 0.059 | 0.102 | 0.535 | 0.204 | 0.418 | 0.068 | 0.186 | 0.058 | 0.040 |
| Sichuan | LSTM | 1.000 | 0.803 | 0.572 | 0.582 | 0.890 | 0.643 | 0.475 | 0.950 | 0.619 | 0.612 | 0.711 | 0.519 | 0.612 | 0.809 | 0.774 |
|  | GM | 0.000 | 0.099 | 0.218 | 0.328 | 0.042 | 0.098 | 0.123 | 0.007 | 0.074 | 0.088 | 0.130 | 0.331 | 0.313 | 0.102 | 0.095 |
|  | ARIMA | 0.000 | 0.098 | 0.210 | 0.090 | 0.068 | 0.259 | 0.402 | 0.044 | 0.307 | 0.300 | 0.159 | 0.149 | 0.075 | 0.089 | 0.131 |
| Guizhou | LSTM | 0.999 | 0.185 | 0.151 | 0.446 | 0.367 | 0.450 | 0.538 | 0.985 | 0.834 | 0.254 | 0.330 | 0.308 | 0.571 | 0.201 | 0.375 |
|  | GM | 0.001 | 0.164 | 0.560 | 0.291 | 0.284 | 0.247 | 0.216 | 0.007 | 0.098 | 0.284 | 0.197 | 0.572 | 0.237 | 0.143 | 0.481 |
|  | ARIMA | 0.000 | 0.651 | 0.289 | 0.263 | 0.349 | 0.303 | 0.246 | 0.008 | 0.069 | 0.462 | 0.473 | 0.120 | 0.192 | 0.657 | 0.144 |
| Yunnan | LSTM | 1.000 | 0.708 | 0.449 | 0.283 | 0.541 | 0.887 | 0.335 | 0.401 | 0.916 | 0.247 | 0.285 | 0.169 | 0.266 | 0.243 | 0.607 |
|  | GM | 0.000 | 0.079 | 0.176 | 0.435 | 0.381 | 0.073 | 0.416 | 0.314 | 0.029 | 0.166 | 0.144 | 0.209 | 0.227 | 0.513 | 0.170 |
|  | ARIMA | 0.000 | 0.213 | 0.374 | 0.282 | 0.078 | 0.040 | 0.249 | 0.285 | 0.055 | 0.586 | 0.572 | 0.623 | 0.507 | 0.244 | 0.223 |
| Tibet | LSTM | 1.000 | 0.743 | 0.014 | 0.613 | 0.546 | 0.907 | 0.751 | 0.867 | 0.860 | 0.901 | 0.847 | 0.034 | 0.065 | 0.038 | 0.628 |
|  | GM | 0.000 | 0.189 | 0.007 | 0.324 | 0.239 | 0.045 | 0.119 | 0.050 | 0.052 | 0.036 | 0.042 | 0.052 | 0.788 | 0.080 | 0.166 |
|  | ARIMA | 0.000 | 0.068 | 0.980 | 0.063 | 0.215 | 0.048 | 0.130 | 0.083 | 0.088 | 0.063 | 0.111 | 0.914 | 0.148 | 0.882 | 0.206 |
| Shaanxi | LSTM | 1.000 | 0.538 | 0.452 | 0.417 | 0.212 | 0.111 | 0.526 | 0.618 | 0.578 | 0.145 | 0.718 | 0.218 | 0.087 | 0.657 | 0.513 |
|  | GM | 0.000 | 0.220 | 0.302 | 0.330 | 0.253 | 0.079 | 0.313 | 0.276 | 0.281 | 0.226 | 0.169 | 0.656 | 0.862 | 0.100 | 0.199 |
|  | ARIMA | 0.000 | 0.242 | 0.246 | 0.252 | 0.534 | 0.809 | 0.161 | 0.105 | 0.140 | 0.629 | 0.113 | 0.126 | 0.052 | 0.243 | 0.288 |
| Gansu | LSTM | 1.000 | 0.591 | 0.377 | 0.262 | 0.315 | 0.984 | 0.460 | 0.406 | 0.574 | 0.608 | 0.503 | 0.302 | 0.310 | 0.742 | 0.687 |
|  | GM | 0.000 | 0.348 | 0.598 | 0.685 | 0.638 | 0.013 | 0.511 | 0.533 | 0.313 | 0.213 | 0.121 | 0.594 | 0.646 | 0.229 | 0.253 |
|  | ARIMA | 0.000 | 0.062 | 0.025 | 0.052 | 0.047 | 0.003 | 0.029 | 0.061 | 0.114 | 0.178 | 0.376 | 0.103 | 0.043 | 0.029 | 0.060 |
| Qinghai | LSTM | 1.000 | 0.511 | 0.509 | 0.653 | 0.847 | 0.773 | 0.791 | 0.133 | 0.445 | 0.089 | 0.044 | 0.380 | 0.578 | 0.392 | 0.434 |
|  | GM | 0.000 | 0.093 | 0.212 | 0.220 | 0.074 | 0.091 | 0.088 | 0.209 | 0.188 | 0.893 | 0.063 | 0.483 | 0.232 | 0.308 | 0.285 |
|  | ARIMA | 0.000 | 0.396 | 0.279 | 0.128 | 0.079 | 0.136 | 0.121 | 0.658 | 0.368 | 0.017 | 0.892 | 0.137 | 0.190 | 0.300 | 0.282 |
| Ningxia | LSTM | 1.000 | 0.765 | 0.397 | 0.820 | 0.285 | 0.845 | 0.983 | 0.122 | 0.697 | 0.649 | 0.341 | 0.337 | 0.227 | 0.255 | 0.365 |
|  | GM | 0.000 | 0.115 | 0.142 | 0.149 | 0.473 | 0.079 | 0.008 | 0.248 | 0.134 | 0.107 | 0.175 | 0.279 | 0.686 | 0.128 | 0.443 |
|  | ARIMA | 0.000 | 0.120 | 0.461 | 0.031 | 0.242 | 0.076 | 0.010 | 0.631 | 0.169 | 0.243 | 0.485 | 0.384 | 0.087 | 0.617 | 0.192 |
| Xinjiang | LSTM | 1.000 | 0.256 | 0.263 | 0.652 | 0.975 | 0.987 | 0.499 | 0.372 | 0.290 | 0.213 | 0.934 | 0.761 | 0.624 | 0.199 | 0.445 |
|  | GM | 0.000 | 0.241 | 0.301 | 0.235 | 0.014 | 0.009 | 0.268 | 0.343 | 0.551 | 0.686 | 0.032 | 0.120 | 0.215 | 0.671 | 0.271 |
|  | ARIMA | 0.000 | 0.503 | 0.436 | 0.114 | 0.011 | 0.004 | 0.233 | 0.285 | 0.158 | 0.102 | 0.034 | 0.119 | 0.161 | 0.129 | 0.283 |

Table S4 The weights of three prediction models from 2022 to 2030

| Province | Model | 2022 | 2023 | 2024 | 2025 | 2026 | 2027 | 2028 | 2029 | 2030 |
| --- | --- | --- | --- | --- | --- | --- | --- | --- | --- | --- |
| Beijing | LSTM | 0.388 | 0.347 | 0.336 | 0.331 | 0.327 | 0.291 | 0.305 | 0.315 | 0.311 |
|  | GM | 0.411 | 0.438 | 0.452 | 0.459 | 0.468 | 0.491 | 0.466 | 0.451 | 0.464 |
|  | ARIMA | 0.201 | 0.214 | 0.212 | 0.210 | 0.205 | 0.218 | 0.229 | 0.234 | 0.225 |
| Tianjin | LSTM | 0.519 | 0.487 | 0.494 | 0.467 | 0.450 | 0.432 | 0.441 | 0.467 | 0.478 |
|  | GM | 0.318 | 0.339 | 0.351 | 0.371 | 0.384 | 0.397 | 0.379 | 0.344 | 0.331 |
|  | ARIMA | 0.163 | 0.174 | 0.155 | 0.162 | 0.166 | 0.171 | 0.180 | 0.190 | 0.191 |
| Hebei | LSTM | 0.461 | 0.425 | 0.416 | 0.430 | 0.435 | 0.433 | 0.417 | 0.405 | 0.390 |
|  | GM | 0.357 | 0.381 | 0.394 | 0.385 | 0.376 | 0.374 | 0.380 | 0.382 | 0.390 |
|  | ARIMA | 0.182 | 0.194 | 0.190 | 0.185 | 0.189 | 0.193 | 0.203 | 0.213 | 0.219 |
| Shanxi | LSTM | 0.422 | 0.383 | 0.376 | 0.379 | 0.388 | 0.409 | 0.420 | 0.423 | 0.424 |
|  | GM | 0.356 | 0.380 | 0.400 | 0.397 | 0.394 | 0.361 | 0.359 | 0.344 | 0.333 |
|  | ARIMA | 0.223 | 0.237 | 0.223 | 0.223 | 0.218 | 0.229 | 0.221 | 0.234 | 0.243 |
| Inner Mongolia | LSTM | 0.374 | 0.332 | 0.351 | 0.369 | 0.357 | 0.355 | 0.353 | 0.371 | 0.350 |
|  | GM | 0.381 | 0.406 | 0.432 | 0.403 | 0.406 | 0.408 | 0.399 | 0.371 | 0.383 |
|  | ARIMA | 0.245 | 0.262 | 0.217 | 0.228 | 0.237 | 0.237 | 0.248 | 0.258 | 0.267 |
| Liaoning | LSTM | 0.402 | 0.362 | 0.374 | 0.371 | 0.345 | 0.327 | 0.331 | 0.346 | 0.339 |
|  | GM | 0.249 | 0.265 | 0.278 | 0.275 | 0.281 | 0.288 | 0.266 | 0.274 | 0.271 |
|  | ARIMA | 0.349 | 0.373 | 0.348 | 0.355 | 0.374 | 0.385 | 0.403 | 0.380 | 0.390 |
| Jilin | LSTM | 0.415 | 0.376 | 0.380 | 0.375 | 0.364 | 0.342 | 0.344 | 0.343 | 0.357 |
|  | GM | 0.322 | 0.343 | 0.351 | 0.356 | 0.364 | 0.376 | 0.371 | 0.379 | 0.354 |
|  | ARIMA | 0.263 | 0.281 | 0.268 | 0.269 | 0.272 | 0.282 | 0.285 | 0.278 | 0.289 |
| Heilongjiang | LSTM | 0.558 | 0.529 | 0.536 | 0.522 | 0.527 | 0.533 | 0.509 | 0.508 | 0.515 |
|  | GM | 0.230 | 0.245 | 0.225 | 0.232 | 0.223 | 0.213 | 0.224 | 0.228 | 0.218 |
|  | ARIMA | 0.212 | 0.227 | 0.239 | 0.246 | 0.250 | 0.254 | 0.267 | 0.265 | 0.266 |
| Shanghai | LSTM | 0.426 | 0.388 | 0.391 | 0.403 | 0.401 | 0.385 | 0.383 | 0.388 | 0.387 |
|  | GM | 0.239 | 0.255 | 0.258 | 0.261 | 0.258 | 0.266 | 0.266 | 0.261 | 0.259 |
|  | ARIMA | 0.335 | 0.357 | 0.350 | 0.336 | 0.341 | 0.350 | 0.352 | 0.351 | 0.354 |
| Jiangsu | LSTM | 0.474 | 0.439 | 0.442 | 0.468 | 0.456 | 0.486 | 0.512 | 0.484 | 0.466 |
|  | GM | 0.387 | 0.413 | 0.414 | 0.440 | 0.451 | 0.415 | 0.384 | 0.406 | 0.419 |
|  | ARIMA | 0.140 | 0.149 | 0.143 | 0.093 | 0.093 | 0.099 | 0.104 | 0.110 | 0.115 |
| Zhejiang | LSTM | 0.481 | 0.446 | 0.462 | 0.450 | 0.446 | 0.466 | 0.476 | 0.485 | 0.494 |
|  | GM | 0.357 | 0.381 | 0.358 | 0.364 | 0.358 | 0.327 | 0.305 | 0.287 | 0.283 |
|  | ARIMA | 0.162 | 0.173 | 0.180 | 0.187 | 0.196 | 0.207 | 0.219 | 0.228 | 0.223 |
| Anhui | LSTM | 0.408 | 0.369 | 0.353 | 0.350 | 0.351 | 0.363 | 0.359 | 0.371 | 0.381 |
|  | GM | 0.272 | 0.290 | 0.306 | 0.318 | 0.315 | 0.293 | 0.292 | 0.278 | 0.261 |
|  | ARIMA | 0.320 | 0.342 | 0.341 | 0.332 | 0.334 | 0.345 | 0.349 | 0.352 | 0.358 |
| Fujian | LSTM | 0.432 | 0.394 | 0.401 | 0.390 | 0.373 | 0.365 | 0.328 | 0.335 | 0.354 |
|  | GM | 0.289 | 0.308 | 0.292 | 0.292 | 0.293 | 0.283 | 0.298 | 0.283 | 0.261 |
|  | ARIMA | 0.279 | 0.298 | 0.307 | 0.318 | 0.334 | 0.351 | 0.374 | 0.382 | 0.385 |
| Jiangxi | LSTM | 0.508 | 0.476 | 0.471 | 0.472 | 0.447 | 0.426 | 0.414 | 0.418 | 0.432 |
|  | GM | 0.307 | 0.327 | 0.333 | 0.329 | 0.344 | 0.357 | 0.365 | 0.358 | 0.347 |
|  | ARIMA | 0.185 | 0.197 | 0.196 | 0.199 | 0.209 | 0.218 | 0.221 | 0.224 | 0.221 |
| Shandong | LSTM | 0.476 | 0.441 | 0.450 | 0.453 | 0.461 | 0.469 | 0.457 | 0.436 | 0.406 |
|  | GM | 0.308 | 0.329 | 0.333 | 0.339 | 0.348 | 0.340 | 0.341 | 0.351 | 0.368 |
|  | ARIMA | 0.215 | 0.230 | 0.217 | 0.208 | 0.191 | 0.191 | 0.203 | 0.213 | 0.226 |
| Henan | LSTM | 0.579 | 0.551 | 0.527 | 0.526 | 0.526 | 0.531 | 0.514 | 0.485 | 0.484 |
|  | GM | 0.256 | 0.273 | 0.290 | 0.295 | 0.290 | 0.281 | 0.292 | 0.310 | 0.320 |
|  | ARIMA | 0.165 | 0.176 | 0.183 | 0.179 | 0.185 | 0.188 | 0.194 | 0.205 | 0.196 |
| Hubei | LSTM | 0.448 | 0.411 | 0.416 | 0.426 | 0.440 | 0.437 | 0.401 | 0.399 | 0.380 |
|  | GM | 0.333 | 0.355 | 0.345 | 0.348 | 0.341 | 0.341 | 0.362 | 0.373 | 0.383 |
|  | ARIMA | 0.219 | 0.234 | 0.239 | 0.227 | 0.218 | 0.223 | 0.237 | 0.228 | 0.236 |
| Hunan | LSTM | 0.533 | 0.502 | 0.516 | 0.529 | 0.501 | 0.478 | 0.487 | 0.498 | 0.520 |
|  | GM | 0.236 | 0.252 | 0.259 | 0.262 | 0.278 | 0.292 | 0.297 | 0.295 | 0.263 |
|  | ARIMA | 0.230 | 0.246 | 0.226 | 0.209 | 0.221 | 0.230 | 0.216 | 0.207 | 0.217 |
| Guangdong | LSTM | 0.489 | 0.454 | 0.436 | 0.452 | 0.454 | 0.459 | 0.469 | 0.493 | 0.500 |
|  | GM | 0.239 | 0.255 | 0.266 | 0.275 | 0.272 | 0.270 | 0.273 | 0.239 | 0.239 |
|  | ARIMA | 0.273 | 0.291 | 0.298 | 0.274 | 0.274 | 0.271 | 0.258 | 0.269 | 0.261 |
| Guangxi | LSTM | 0.483 | 0.449 | 0.452 | 0.460 | 0.465 | 0.467 | 0.467 | 0.461 | 0.438 |
|  | GM | 0.285 | 0.304 | 0.291 | 0.295 | 0.297 | 0.302 | 0.302 | 0.310 | 0.322 |
|  | ARIMA | 0.232 | 0.248 | 0.257 | 0.245 | 0.238 | 0.231 | 0.231 | 0.229 | 0.240 |
| Hainan | LSTM | 0.454 | 0.417 | 0.443 | 0.444 | 0.459 | 0.434 | 0.409 | 0.393 | 0.393 |
|  | GM | 0.277 | 0.296 | 0.313 | 0.317 | 0.325 | 0.339 | 0.353 | 0.361 | 0.359 |
|  | ARIMA | 0.269 | 0.287 | 0.244 | 0.239 | 0.216 | 0.227 | 0.238 | 0.245 | 0.248 |
| Chongqing | LSTM | 0.738 | 0.721 | 0.726 | 0.712 | 0.714 | 0.714 | 0.708 | 0.694 | 0.684 |
|  | GM | 0.114 | 0.122 | 0.118 | 0.124 | 0.115 | 0.114 | 0.117 | 0.122 | 0.127 |
|  | ARIMA | 0.147 | 0.157 | 0.156 | 0.165 | 0.170 | 0.172 | 0.176 | 0.184 | 0.189 |
| Sichuan | LSTM | 0.705 | 0.685 | 0.677 | 0.684 | 0.691 | 0.678 | 0.680 | 0.694 | 0.677 |
|  | GM | 0.137 | 0.146 | 0.149 | 0.144 | 0.132 | 0.138 | 0.141 | 0.142 | 0.151 |
|  | ARIMA | 0.159 | 0.169 | 0.174 | 0.172 | 0.177 | 0.184 | 0.179 | 0.165 | 0.173 |
| Guizhou | LSTM | 0.466 | 0.431 | 0.447 | 0.467 | 0.468 | 0.475 | 0.477 | 0.473 | 0.438 |
|  | GM | 0.252 | 0.269 | 0.276 | 0.257 | 0.255 | 0.253 | 0.253 | 0.255 | 0.272 |
|  | ARIMA | 0.282 | 0.300 | 0.277 | 0.276 | 0.277 | 0.272 | 0.270 | 0.272 | 0.290 |
| Yunnan | LSTM | 0.489 | 0.455 | 0.438 | 0.437 | 0.448 | 0.442 | 0.412 | 0.417 | 0.418 |
|  | GM | 0.222 | 0.237 | 0.248 | 0.252 | 0.240 | 0.231 | 0.241 | 0.230 | 0.224 |
|  | ARIMA | 0.289 | 0.308 | 0.314 | 0.310 | 0.312 | 0.328 | 0.347 | 0.353 | 0.358 |
| Tibet | LSTM | 0.588 | 0.560 | 0.548 | 0.584 | 0.582 | 0.584 | 0.562 | 0.550 | 0.529 |
|  | GM | 0.146 | 0.156 | 0.153 | 0.163 | 0.152 | 0.147 | 0.153 | 0.156 | 0.163 |
|  | ARIMA | 0.267 | 0.284 | 0.299 | 0.253 | 0.266 | 0.269 | 0.284 | 0.294 | 0.309 |
| Shaanxi | LSTM | 0.453 | 0.416 | 0.408 | 0.405 | 0.405 | 0.417 | 0.438 | 0.432 | 0.419 |
|  | GM | 0.284 | 0.303 | 0.309 | 0.309 | 0.308 | 0.312 | 0.327 | 0.328 | 0.332 |
|  | ARIMA | 0.263 | 0.280 | 0.283 | 0.285 | 0.287 | 0.271 | 0.235 | 0.240 | 0.249 |
| Gansu | LSTM | 0.542 | 0.511 | 0.506 | 0.514 | 0.531 | 0.545 | 0.516 | 0.520 | 0.527 |
|  | GM | 0.380 | 0.405 | 0.409 | 0.396 | 0.377 | 0.360 | 0.383 | 0.374 | 0.364 |
|  | ARIMA | 0.079 | 0.084 | 0.085 | 0.089 | 0.092 | 0.095 | 0.101 | 0.106 | 0.109 |
| Qinghai | LSTM | 0.505 | 0.472 | 0.470 | 0.467 | 0.455 | 0.429 | 0.406 | 0.380 | 0.396 |
|  | GM | 0.229 | 0.245 | 0.255 | 0.257 | 0.260 | 0.272 | 0.284 | 0.298 | 0.303 |
|  | ARIMA | 0.265 | 0.283 | 0.276 | 0.275 | 0.285 | 0.299 | 0.310 | 0.323 | 0.300 |
| Ningxia | LSTM | 0.539 | 0.508 | 0.491 | 0.498 | 0.476 | 0.489 | 0.465 | 0.431 | 0.451 |
|  | GM | 0.211 | 0.225 | 0.233 | 0.239 | 0.245 | 0.229 | 0.239 | 0.255 | 0.255 |
|  | ARIMA | 0.250 | 0.266 | 0.276 | 0.264 | 0.279 | 0.282 | 0.296 | 0.315 | 0.294 |
| Xinjiang | LSTM | 0.565 | 0.536 | 0.554 | 0.574 | 0.569 | 0.541 | 0.512 | 0.513 | 0.522 |
|  | GM | 0.264 | 0.281 | 0.284 | 0.283 | 0.286 | 0.304 | 0.324 | 0.328 | 0.327 |
|  | ARIMA | 0.171 | 0.183 | 0.162 | 0.143 | 0.145 | 0.154 | 0.164 | 0.160 | 0.151 |
